# Deleterious variants in *LTBP4* are associated with severe pediatric sepsis

**DOI:** 10.1038/s41390-025-04420-3

**Published:** 2025-10-11

**Authors:** Yidi Qin, Kate F. Kernan, Yulong Bai, John R. Shaffer, Zsolt Urban, Scott Canna, Murray M. Pollack, Kathleen Meert, Christopher Newth, Tom Shanley, Rick E. Harrison, Mark Hall, Joseph A. Carcillo, Hyun-Jung Park

**Affiliations:** 1 Department of Human Genetics, School of Public Health, University of Pittsburgh, Pittsburgh, PA, USA.; 2 Division of Pediatric Critical Care Medicine, Department of Critical Care Medicine, Children’s Hospital of Pittsburgh, Center for Critical Care Nephrology and Clinical Research Investigation and Systems Modeling of Acute Illness Center, Faculty Pavilion, UPMC Children’s Hospital of Pittsburgh, University of Pittsburgh, Pittsburgh, PA, USA.; 3 Center for Craniofacial and Dental Genetics, Department of Oral and Craniofacial Sciences, School of Dental Medicine, University of Pittsburgh, Pittsburgh, PA, USA.; 4 Department of Pediatrics, Children’s Hospital of Philadelphia, Immune Dysregulation Program, Division of Rheumatology, University of Pennsylvania School of Medicine, Pittsburgh, PA, USA.; 5 Division of Critical Care Medicine, Department of Pediatrics, Children’s National Hospital, Washington DC, USA.; 6 Division of Critical Care Medicine, Department of Pediatrics, Children’s Hospital of Michigan, Detroit, MI, USA.; 7 Central Michigan University, Mt. Pleasant, MI, USA.; 8 Division of Critical Care Medicine, Department of Anesthesiology and Critical Care Medicine, Children’s Hospital Los Angeles, Los Angeles, CA, USA.; 9 Division of Critical Care Medicine, Department of Pediatrics, C. S. Mott Children’s Hospital, Ann Arbor, MI, USA.; 10 Division of Critical Care Medicine, Department of Pediatrics, Mattel Children’s Hospital at University of California Los Angeles, Los Angeles, CA, USA.; 11 Division of Critical Care Medicine, Department of Pediatrics, The Research Institute at Nationwide Children’s Hospital Immune Surveillance Laboratory, and Nationwide Children’s Hospital, Columbus, OH, USA.

## Abstract

**BACKGROUND:**

Sepsis is a leading global health burden in children, and its unavoidable heterogeneity has hindered providing therapies beyond antibiotics and supportive care. Recently, we identified four computable phenotypes showing distinct cytokine profiles, clinical outcomes, and therapeutic response characteristics (PedSep-A, B, C, and D) in a multicenter pediatric sepsis cohort.

**METHODS:**

In the cohort data, we collected whole-exome sequencing data and identified rare variants associated with PedSep-D phenotype by conducting a gene-based analysis in an aggregated fashion.

**RESULTS:**

As a result, one whole-exome significant gene (*LTBP4*) and two suggestive significant genes (*PLA2G4E*, *CCDC157*) showed association with PedSep-D, the phenotype characterized by the most severe outcomes and highest inflammation. The associated variants in *LTBP4* were enriched for predicted deleterious effects based on established functional prediction metrics. All three associated genes are implicated in inflammation and immune cell activation based on existing gene function and expression data. Although the circulating cytokine profiles were overlapping between the rare variant carriers, we also identified gene-specific cytokine changes.

**CONCLUSION:**

Altogether, our study provides valuable insights into the genetic architecture of a pediatric sepsis phenotype with the highest inflammation level and the most severe outcomes, highlighting potential candidate genes and pathways for further biomarker and therapeutic studies.

## INTRODUCTION

Pediatric sepsis is a life-threatening condition associated with organ failure in children due to a dysregulated host immune response to infection. It is a recognized global public health problem that affects 20.3 million children and causes 2.9 million deaths in those under five years old every year.^1^ Despite global efforts to improve clinical outcomes for pediatric sepsis, its phenotypic heterogeneity remains a significant barrier to therapeutic advancement.^2^ Several recent studies have advanced efforts to characterize pediatric sepsis phenotypes using data-driven approaches, including genome-wide expression profiling,^3,4^ dynamic modeling of organ dysfunction trajectories,^5^ and supervised classification of inflammation-based subtypes.^6^ While these contributions are important, key limitations remain. Studies, such as Wong et al.^3^ and Sweeney et al.^4^ primarily rely on transcriptomic data, which, although informative at the molecular level, may lack direct applicability at the bedside. While Sanchez-Pinto et al.^5^ utilized subscores of the pediatric Sequential Organ Failure Assessment (pSOFA) score to define multiple organ dysfunction syndrome (MODS) phenotypes, these phenotypes may not fully reflect the broader complexity and heterogeneity of pediatric sepsis, particularly features not directly manifested as organ failure. Although Carcillo et al.^6^ included a broader range of clinical features, their supervised approach introduces the potential for bias due to dependence on predefined categories or outcomes. In contrast, our approach aims to address these limitations by leveraging a broader and more granular set of clinical data in an unsupervised framework that emphasizes both statistical robustness and bedside applicability. Recently, by applying machine learning approaches to 25 first-day bedside clinical variables of 404 pediatric sepsis patients with organ dysfunction enrolled as part of a multicenter cohort, PHENOtyping sepsis-induced Multiple organ failure Study (PHENOMS), between 2015 to 2017, we derived four computable phenotypes PedSep-A, B, C, and D with differences in infection source, cytokine profiles, organ failure, outcomes, and treatment responses.^7^

Several studies suggested that host genetic factors contribute to the heterogeneity of pediatric sepsis. While early family studies^8^ and targeted candidate gene analyses^9–11^ have supported this notion, discovery efforts have been limited to discover novel functional sepsis-related genes. Alternatively, researchers have conducted several genome-wide association studies (GWAS) on adult and pediatric populations to identify common variants underlying sepsis susceptibility and outcomes.^12–14^ However, although common variants have been used to understand the genetic basis of clinical outcomes (e.g., survival from sepsis or hospital admissions), they are limited in elucidating the genetic architecture for computable pediatric sepsis phenotypes with poor outcomes. Since common variants usually have small effects on complex traits, their systematic study requires a prohibitively large sample size and their small effects are of limited clinical benefit in a large fraction of the population, which is not feasible to study in pediatric sepsis.^15,16^ Additionally, to the best of our knowledge, studies investigating rare variants (low penetrance, low allele frequency (AF)) in pediatric critical illness are limited,^17^ with no prior rare variant analyses specifically focusing on sepsis subtypes. This gap underscores the need for more targeted genomic investigations to identify genetic contributors to pediatric sepsis severity and heterogeneity. These findings underscore the need for more comprehensive and functionally focused genomic studies, especially in understudied pediatric populations. Without involving further functional validation, the large fraction of findings in non-coding regions challenges the interpretation of the sepsis GWAS results.

**Fig. 1:**
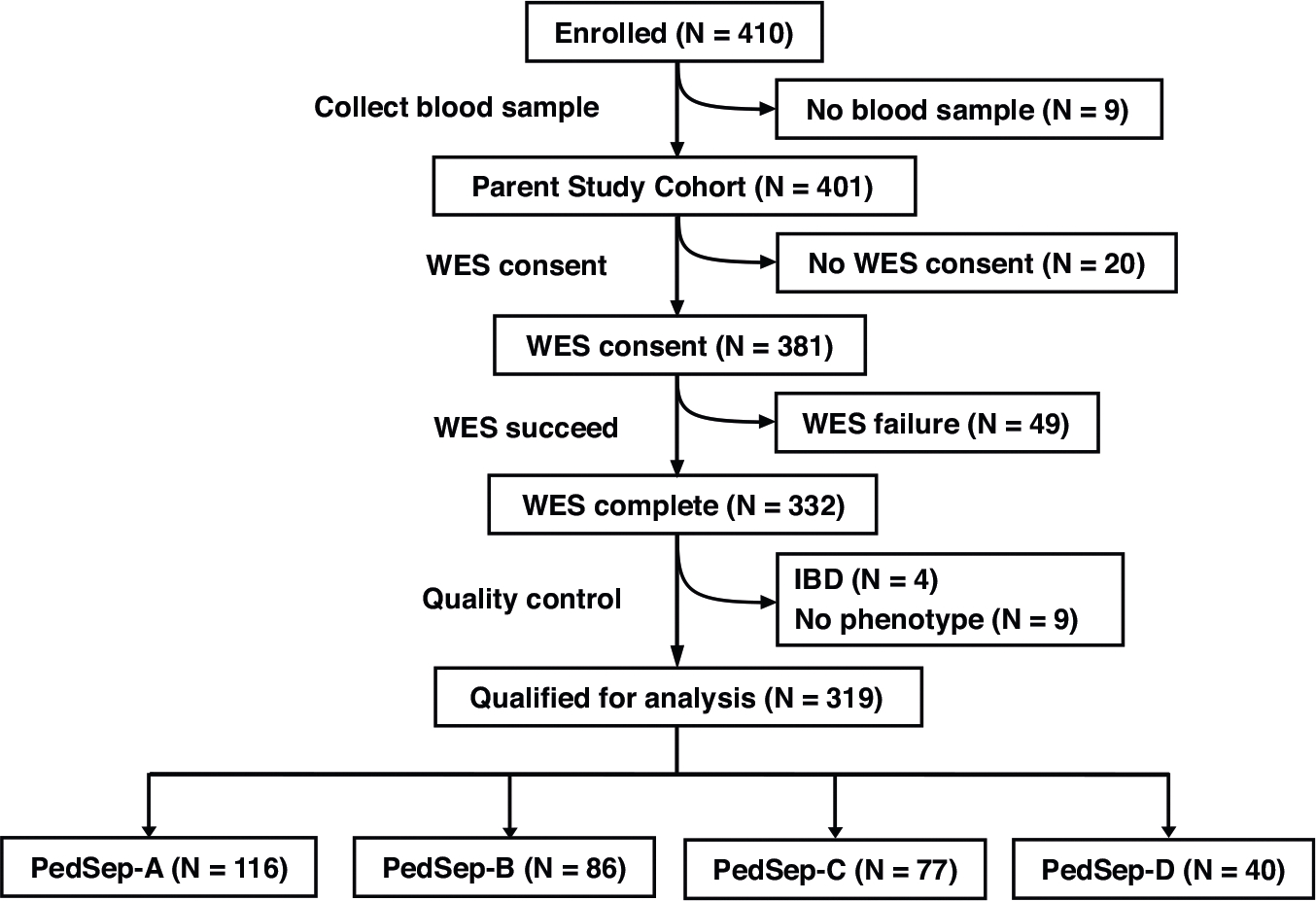
CONSORT (Consolidated Standards of Reporting Trials) diagram for the study.

To address these limitations, we performed a gene-based exome-wide rare variant analysis using data from the PHENOMS study of severe pediatric sepsis. Using whole-exome sequencing data from 319 children (Fig. 1) with sepsis and organ dysfunction in the PHENOMS study, we focused our analysis on the highest-risk phenotype, PedSep-D, with the aim of identifying biologically impactful variants and informing future therapeutic strategies. Altogether, we present the first rare variant burden test in the computable phenotype with the worst outcomes in pediatric sepsis.

## METHODS AND MATERIALS

### Consent statement

The study was approved by the Institutional Review Board at University of Utah Central IRB # 70976.

### Cohort and phenotyping

The phenotyping data and blood samples were obtained from pediatric sepsis patients of a multicenter cohort, PHENOMS.^6^ The cohort enrolled pediatric patients from 2015 to 2017 with written informed consent from at least one of the guardians. Children were qualified for enrollment if they met all four criteria: (1) at the ages of 44 weeks to 18 years old; (2) were suspected of infection meeting two or more SIRS (systemic inflammatory response) criteria;^18^ (3) presented one or more organ failures; and (4) had an indwelling arterial or central venous catheter. Patients without a commitment to aggressive care or lack of blood samples were further excluded from the enrollment.

The data-driven phenotyping approach and results were described in previous work.^7^ Briefly, four phenotypes named PedSep-A, B, C, and D were identified by applying consensus k-means clustering on 25 day-one bedside variables. As WES data was available for only a subset of the complete cohort, we confirmed the phenotyping analysis using this subset of patients with available genetic data to ensure that phenotype assignment was robust in this reduced sample size. In the following genetic analysis, we performed a case-control study between children in the computable phenotype subgroup with the highest severity of illness and mortality (PedSep-D) and the others (PedSepA,B, and C) to identify the genetic factors exclusively associated with increased sepsis severity susceptibility.

### DNA extraction and genotyping

Out of 404 pediatric patients enrolled in the cohort, a total of 381 parents of the children provided WES consent, and 2 mL of whole blood was collected for DNA extraction using standard methods. Whole-exome sequencing was successfully completed on 332 patients from 2018 to 2020 by the University of Pittsburgh Genomics Research Core performed on the Ion Torrent platform. Libraries were constructed by the Ampliseq Exome RDY (Thermo Fisher Scientific) with 100 × target coverage. FASTQ files were aligned to Homo sapiens reference sequence GRCh37/hg19 to generate VCF files. Variant calling was performed by GATK (Genome Analysis Toolkit).^19^

### Quality control

Two levels of quality control were conducted on 332 samples with completed whole-exome sequencing data, patient-level, and variant-level. At the patient level, we excluded nine individuals without phenotype information. Four pairs of individuals were identified as relatives based on IBD (identity by descent). In each IBD pair, the individual with the higher missingness was removed from the analysis. In terms of variant-level quality control, we filtered sites with SOR (Strand Odds Ratio) > 3, MQ (root mean square Mapping Quality) < 40, QD (variant confidence normalized by depth) < 2.0, average GQ (Genotyping confidence) < 20, average DP (Depth) < 10, missingness > 0.05, HWE (Hardy-Weinberg equilibrium p) < 1e-06, and those located in sex chromosomes. No imputation of missing genotypes was performed due to concerns for potentially low imputation quality of rare variants in datasets with small sample sizes. Quality control was performed by software bcftools (v1.9),^20^ VCFtools (v0.1.16),^21^ and PLINK (v1.9).^22^ Then variant function was annotated by ANNOVAR.^23^

### Principal Component (PC) derivation

To account for potential population stratification and other confounders in the statistical model, we derived 10 principal components (PCs) based on common SNPs following linkage disequilibrium (LD) pruning. LD pruning was performed using PLINK (version 1.07) with the argument “–indep-pairwise 50 5 0.2”. This procedure involves considering a sliding window of 50 SNPs, calculating LD between each pair of SNPs within this window, and removing one SNP from any pair exhibiting an LD greater than 0.2. After pruning within a window, the window is shifted forward by 5 SNPs, and the pruning process is repeated until the entire dataset is processed.

### Gene-based analysis

Variants that passed quality control were included if they were in hg19 annotated exon regions and had a MAF (minor AF) lower than 1%. Genes with less than three qualified variants were excluded from the analysis. The final number of genes tested was 3846. Therefore, the p-value threshold for declaring whole-exome level significance was 0.05/3846 = 1.3e-05.

Then, we aggregately examined the relationships between the rare variants and the binary indicator of phenotype membership by gene-based association test SKAT (Sequence Kernel Association Test).^24^ SKAT is a widely-employed method to test the association between a group of variants and the trait, which increases the power to detect rare variant associations by pooling rare variants across a given region of interest, such as chromosome region or gene. In running the SKAT test, a single null model was fitted containing only the covariates to be adjusted (i.e., age, sex, and the first four ancestry PCs constructed from common LD-pruned SNPs). Then the effect of SNPs from each gene was tested by variance-component score tests in a mixed model, and their statistics were aggregated with weights through a kernel matrix to form a gene-level statistic. Compared to other gene-based tests, such as the Burden Test and SKAT-O, one advantage of applying SKAT in our analysis is that it makes few assumptions about rare-variant effects and retains statistical power when variants within a gene have different directions and magnitude of effects.^25^ This property aligns with the study design that contrasts one phenotype with others and allows us to better account for potential heterogeneity in phenotypes.

Genes showing whole-exome level significance and suggestive significance were further investigated to query the gene function (GeneCards),^26^ common variant evidence from previous GWAS analysis (GWAS Catalog),^27^ gene enrichment in GO biological process (FUMA, Enrichr),^28,29^ and gene expression level in the GTEx database.^30^ Rare variants that contributed to gene significance were annotated with four different types of score (CADD,^31^ GERP,^32^ SIFT,^33^ Polyphen2^34^) to indicate the effect of each variant.

### Comparison of cytokine profiles between rare variant carriers and non-carriers

To further investigate the effect of variations on inflammation, levels of the 33 pre-collected biomarkers of the rare variant carriers were further visualized and compared with non-carriers. The cytokine heatmap was used to present the log ratio of the median biomarker values of the host response. The red color represents a greater value for the group compared to the entire cohort, while the blue color represents a lower value for the group compared to the entire cohort. Hierarchical clustering was used to visualize the similarity of cytokine patterns between rare variant carriers. Additionally, we calculated p-values from a pairwise t-test comparing cytokine values of rare variant carriers and non-carriers.

### Mediation Analysis

To further explore potential biological mechanisms, we conducted a causal mediation analysis to evaluate whether biomarkers mediate the relationship between rare variant gene burden and PedSep-D membership. Mediation analysis requires three conditions to be met: 1) A significant association between gene burden and PedSep-D membership (which was established by SKAT results); 2) A significant association between gene burden and the candidate biomarker; and 3) A significant association between the biomarker and PedSep-D membership.

When all three conditions were satisfied, we used the mediation package in R,^35^ applying 1000 bootstrap iterations, to estimate the indirect effect (via the biomarker), direct effect, total effect, and proportion mediated for each gene–biomarker pair.

### Sensitivity Analysis

In order to further validate the top genes identified from the gene-based analysis, we investigated the influence of the ancestry information on the genes with a sensitivity analysis that was performed as follows. Briefly, we randomly swap phenotype labels between pairs of individuals with the same reported ancestry information to keep the ancestry makeup of the groups the same and run 100,000 iterations of SKAT gene-based analysis to calculate how many times the test statistic value is greater than the test statistic value from observed data. Thus, we generated an empirical p-value for each gene.

### Pathway-based analysis

In addition to identifying association signals between individual genes and the sepsis phenotype of interest, we also sought to identify associations between genetic variation at the pathway level, using Gene set analysis Association Using Sparse Signals (GAUSS).^36^ GAUSS is constructed with gene-based test results and calculates a gene set level p-value by identifying a subset of genes (i.e., core genes) to maximize the association signal. Using the GAUSS method, we aggregated the SKAT test statistics of individual genes into groups based on GO biological process (GOBP) pathway annotations and examined the associations between 7482 GOBP pathways and phenotype of interest. This was performed for the PedSep-D phenotype (Table 1). We also detected the active genes driving the pathway-trait associations to facilitate the interpretation of test results.

## RESULTS

### PedSep-D phenotype has the highest mortality with unique clinical presentation and immune system profile

**Fig. 2:**
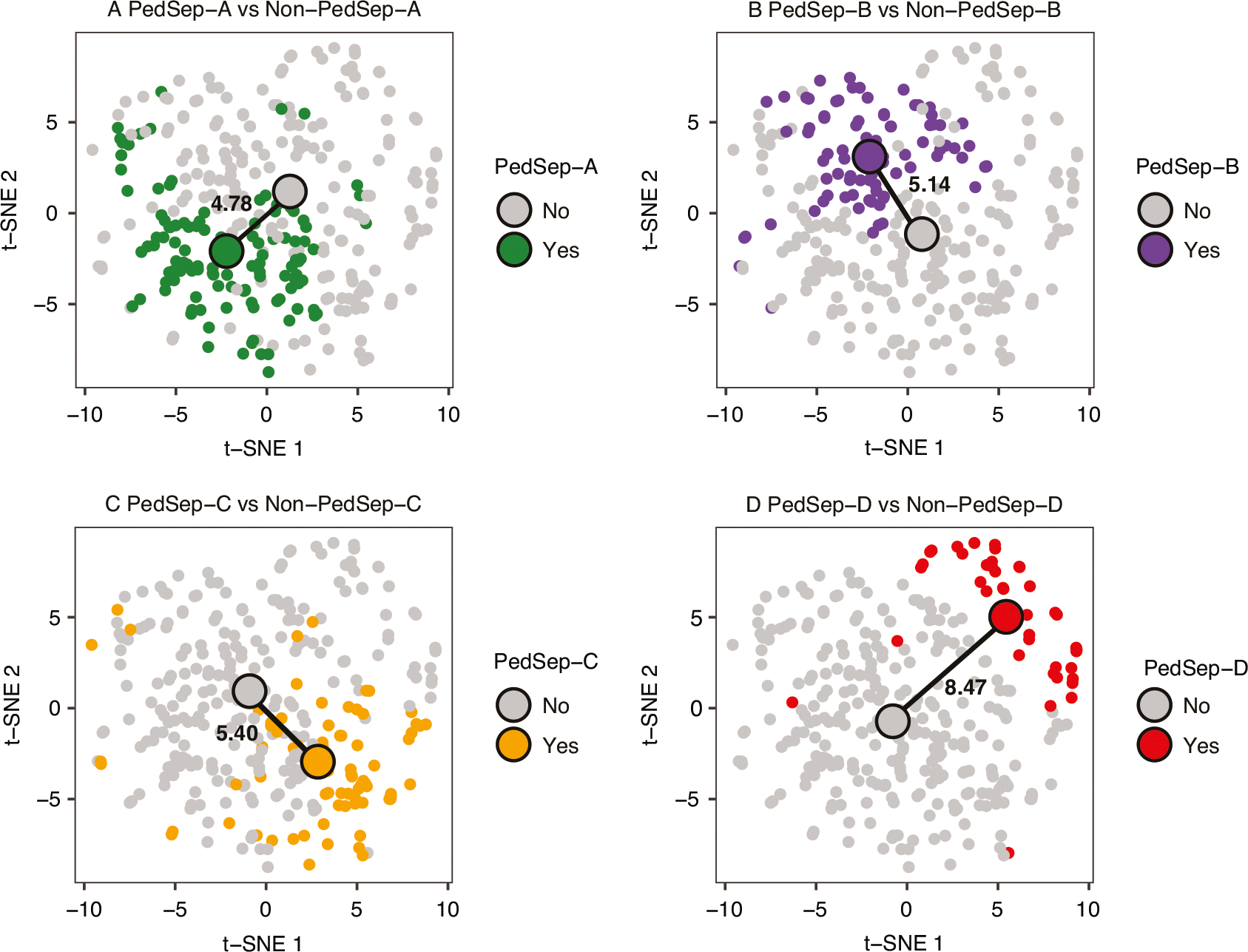
t-SNE plot of four phenotypes (N = 319). Samples in two-dimensional space were labeled by PedSep phenotype membership. Euclidean distance was measured between centroids of two groups to quantitatively compare the difference between one phenotype versus the remaining phenotypes.

Using the 319 pediatric patients from the parent cohort who passed quality control both in bedside features and whole exome sequencing (WES) data (Fig. 1), we assigned them to one of four established phenotypes (PedSep-A, B, C, D) as previously determined by the consensus k-means clustering of 25 first-day bedside features.^7^ Among these 319 patients, the sample sizes of PedSep-A, B, C, D are 116 (36%), 86 (27%), 77 (24%), and 40 (13%), respectively (Table 2, Supplemental Tables 1–3). The proportions of patients in each phenotype are close to our original study, in which PedSep-A, B, C, D contained 34, 25, 27, and 14 percent of the 404 patients, respectively. To estimate the homogeneity of each phenotype, we projected them on the t-SNE plot of all 25 features and observed good separation between each phenotype and the other phenotypes (average Euclidean distance=6.4, Fig. 2). Specifically, the PedSep-D phenotype was distinct from PedSep-A, B, and C patients (Euclidean distance = 8.9).

Our data, which is sampled from the original PHENOMS cohort, confirmed distinct clinical (Table 2; Supplemental Tables 1–3) and biomarker (Supplemental Tables 4–7) profiles across phenotypes. PedSep-A showed the mildest presentation (Supplemental Tables 1 and 4) and PedSep-D had the most severe profile with multi-organ failure and highest mortality (Tables 2–4), where PedSep-B and -C are in bet-ween (Supplemental Tables 2 and 6). These patterns recapitulate the importance of analyzing PedSep-D as the highest-risk group (Supplemental Table 8).

### Gene-based test associates *LTBP4*, *PLA2G4E*, and *CCDC157* with PedSep-D

**Fig. 3:**
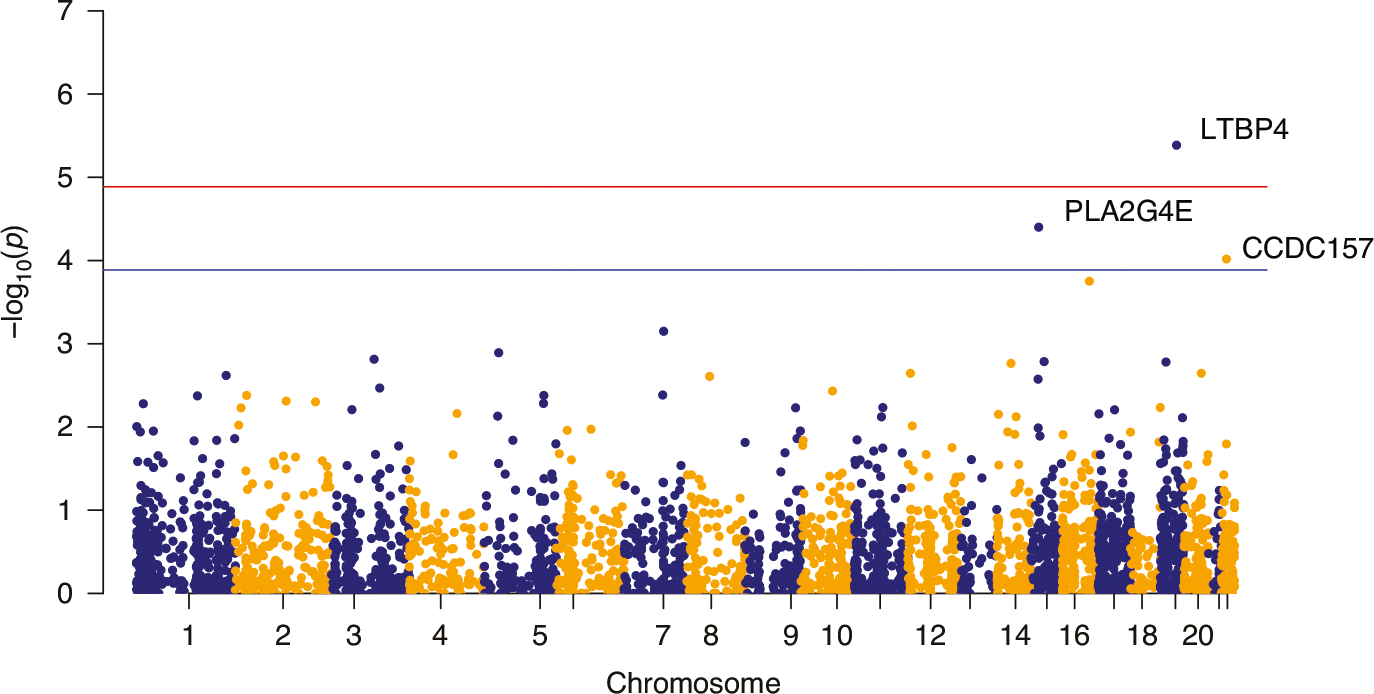
Manhattan plot for PedSep-D (No. of genes = 3846). Red line: whole-exome wide significant −log10(P) value; Blue line: suggested significant −log10(P) value.

To detect genetic factors associated with the sepsis phenotypes, we performed a whole exome-wide rare variant analysis. To increase power in detecting associations, we aggregated the rare variant association signals by gene and estimated the significance in the following steps (see Methods). First, we performed quality control and selected a total of 3864 genes that had more than three variants for the association between rare variants and the phenotype of interest. Then, we ran SKAT on the WES data separately for each of the four PedSep phenotypes (PedSep-A, B, C, D) versus any of the other three phenotypes while adjusting for age, sex, and ancestry based upon the first four PCs constructed based on common variants. (Fig. 3, Supplemental Figs. S1–3, Tables 2, 3, Supplemental Table 9).

**Fig. 4:**
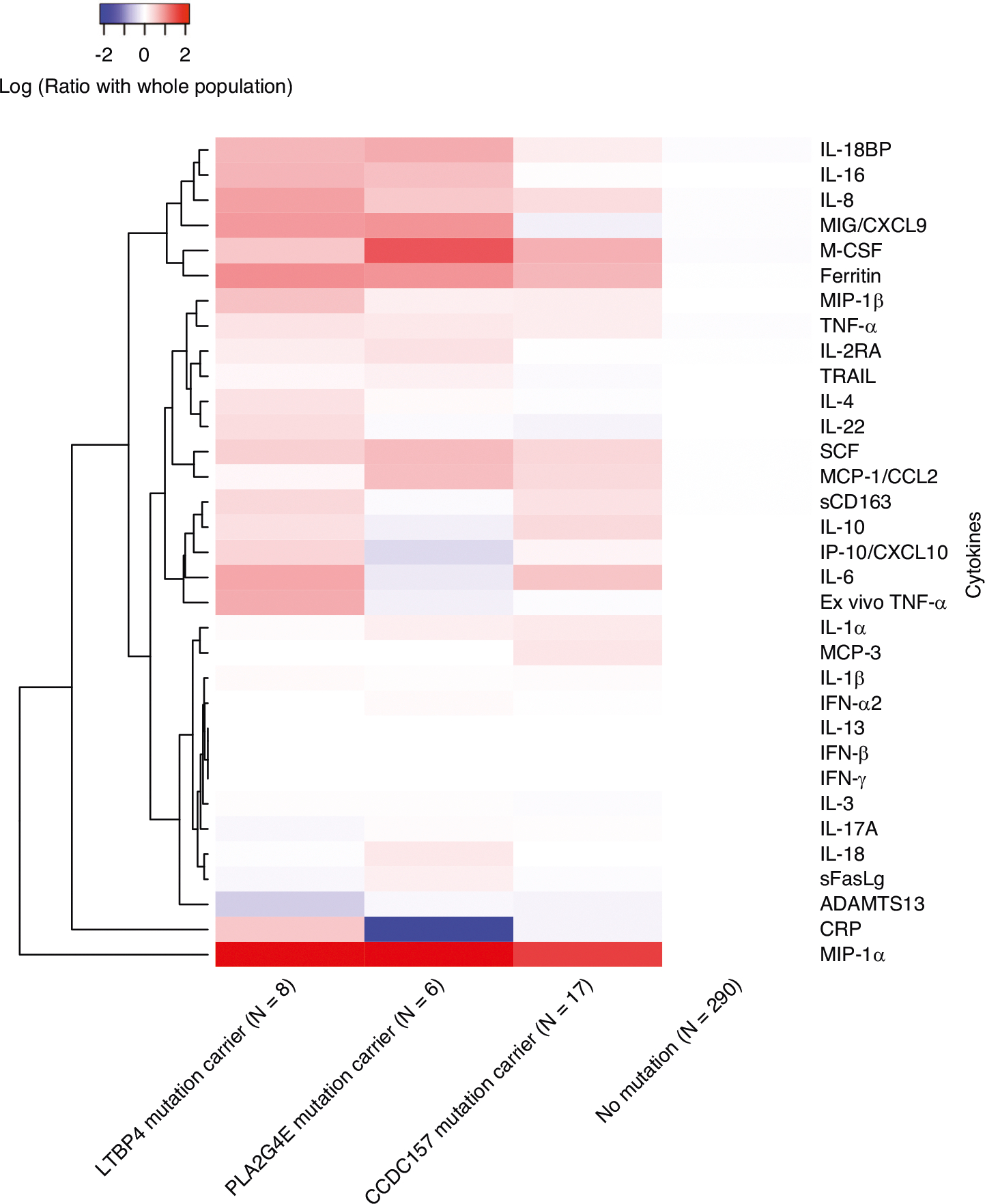
Biomarker heatmap of three genes’ carriers and non-carriers. The log ratio of the median values of 33 inflammatory biomarkers by rare variants carriers and non-carriers. Red represents a greater median biomarker value for that group compared with the median for the entire study cohort, whereas blue represents a lower median biomarker value compared with the median for the entire study cohort.

While our primary analyses centered on PedSep-D, we included results from the other phenotype comparisons to provide broader context and to highlight the distinctiveness of PedSep-D-specific genetic associations. For PedSep-A or C versus the remaining phenotypes, no significantly associated genes were detected. For PedSep-B, the *PLXNA2* gene presented a suggestive association (Supplemental Fig. S2). However, the QQ plot (S. Fig. 4B) showed genomic inflation, suggesting that the potential association of *PLXNA2* has a high risk of being false positive. In contrast, for PedSep-D, variation in *LTBP4* was significantly associated with phenotype development at the exome-wide level (p-value = 1.069 E-05, 6 [15%] carriers in case group, 2 [0.7%] carriers in control group), while variations in *PLA2G4E* (4 [10%] carriers in case group, 2 [0.7%] carriers in control group) and *CCDC157* (7 [17.5%] carriers in case group, 10 [3.6%] carriers in control group) were suggestively associated with phenotype development. Four, 4, and 8 rare variants contributed to the significance of *LTBP4*, *PLA2G4E*, and *CCDC157*, respectively (Table 5). All variants encode missense variants except one in the *LTBP4* gene. However, this silent variant, rs370696272, replaces a common leucine code (CTG, 0.361) with a less common codon (TTG, 0.134) based on the CoCoPUT database,^37^ explaining its high CADD score (17.55). Most variants in three genes were predicted to be deleterious based on their CADD score (12 out of 16 with CADD > 10), among which SNP rs573310430 in *LTBP4* had the highest CADD score of 34, ranked over the top 0.1% in terms of deleteriousness among variants across the whole genome. This variant creates an unpaired cysteine in the 14th calcium-binding epidermal growth factor-like (cbEGF) domain of *LTBP4*, a domain stabilized by three pairs of cysteines forming intradomain disulfide bonds particularly sensitive to removal or addition of cysteine residues.^38^

We observed well-calibrated test statistics and little evidence of inflation (Supplemental Fig. S4, lambda = 0.98) for PedSep-D, suggesting that these associations are true signals. To explore the AF of variants contributing to significant and suggestive genes, we compared the AF of all variants across three populations (Black, White, and Asian) in the gnomAD database (Supplemental Table 9). No large difference was observed between the AFs across populations, indicating that the top signals are less likely related to ancestry distinctions. To further investigate if there is an ancestry difference driving the top signals, we conducted a sensitivity analysis by randomly swapping the phenotype labels between pairs of individuals with similar ancestry information to keep the ancestry makeup of the groups the same while generating a meaningful empirical p-value. With 100,000 iterations of permutation for each of the three genes, we observed 0 times that permutated statistics were larger than the previously estimated statistic. This indicates the significance of the three genes is not likely driven by ancestry differences.

### *LTBP4*, *PLA2G4E*, and *CCDC157* underlie distinct cytokine patterns in patients

To explore the genes’ association with inflammation status, we grouped patients based on whether they carried rare variants in one of the three genes of interest, generated a heatmap showing the normalized levels of 33 cytokines (Fig. 4), and statistically tested the group-wise differences (Supplemental Tables 10, 11). Comparison between the rare variants carriers and non-carriers indicated some similarity shared by carriers groups. For example, a higher level of IL-6 is significantly related to both *LTBP4* and *CCDC157* rare variant carriers (p-value = 0.032 and 0.043, respectively), and higher level of M-CSF is significantly related to both *PLA2G4E* and *CCDC157* rare variant carriers (p-value = 0.013 and 0.011 separately). Simultaneously, several cytokines important in regulating inflammation showed distinct patterns when comparing carriers to non-carriers with rare variants in *LTBP4*, *PLA2G4E*, and *CCDC157* (Supplemental Table 13). For instance, IL-4 is significantly higher in *LTBP4* rare variant carriers (p-value = 0.025) but is significantly lower in *CCDC157* rare variant carriers (p-value = 0.035). Compared to non-carriers, *PLA2G4E* rare variant carriers presented significantly higher levels of IL-16, and SCF, but significantly lower levels of CRPH (p-value = 0.034, 0.005, and 0.022, separately), while *LTBP4* and *CCDC157* rare variant carrier groups showed no significant difference with non-carriers for these biomarkers. The ferritin level is uniquely higher in *LTBP4* rare variant carriers (p-value = 0.023). These results imply that the three genes might be involved in different pathological mechanisms driving the phenotype.

Mediation Analysis was further performed to evaluate whether biomarkers mediate the relationship between rare variant gene burden and PedSep-D membership. The results (Supplemental Table 12) highlight three gene–biomarker pairs that showed significant mediation effects. For example, the effect of LTBP4 burden on PedSep-D membership was partially mediated by ADAMTS13 levels, with 16% of the total effect explained by this biomarker. Similarly, IL-16 and IL-8 were significant mediators for PLA2G4E and CCDC157, respectively, with mediation proportions ranging from 16 to 41%. These findings suggest that biomarkers may serve as functional intermediates linking rare variant burden to sepsis subtype PedSep-D and highlight potential targets for mechanistic validation.

**Fig. 5:**
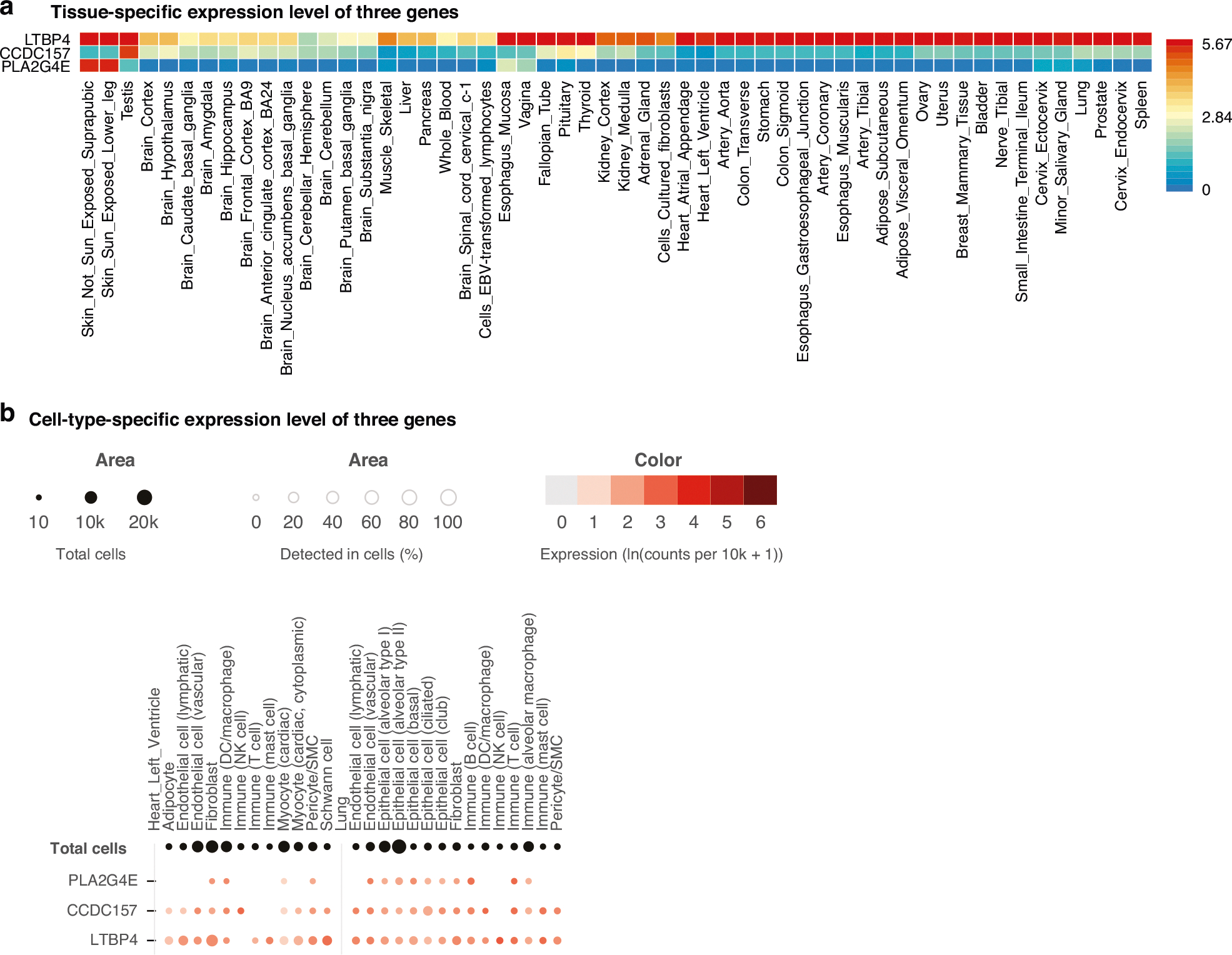
Expression of three genes in different tissues and cell types. **a** Heat map of tissue-specific log2 transformed average expression level for three genes based on GTEx v8 RNA data. Red color indicates a higher expression within a tissue compared to other tissues, whereas blue color indicates a lower expression within a tissue compared to other tissues. **b** Dot plot of cell-type-specific expression level for three genes in two sepsis-related tissues (GTEx Single Cell data). The dot color reports the mean expression value with each cell type. The dot size reports the fraction of cells in which a gene is detected. Black dot size reports the total detected level of all three genes.

In addition, we accessed tissue-specific gene expression data from the GTEx database. Given that GTEx compiles transcriptomic profiles across a wide range of human tissues, we observed that all three genes are expressed in multiple tissue types (Fig. 5a). *LTBP4* is highly expressed in multiple tissues, including the lung, kidney, stomach, skin, and others. *PLA2G4E* is specifically expressed in the skin. *CCDC157* is specifically expressed in testis. In terms of cell-type specific expression of three genes, all of them displayed expression in immune cells of the cardiovascular and pulmonary systems, both of which are highly affected by severe sepsis or septic shock (Fig. 5b).

## DISCUSSION

In this study, rare variants in *LTBP4* were significantly associated with the development of the previously reported high-mortality PedSep-D phenotype, with additional suggestions of associations with rare variations in *PLA2G4E* and *CCDC157*. To our knowledge, this is the first time a rare variant burden test has been applied to pediatric sepsis with deep phenotyping.

The top signal found in our study, *LTBP4*, a member of the latent transforming growth factor β binding protein family, shares structural homology with fibrillin and is moderately expressed in plasma cells and immune cells.^39^ Mutations in *LTBP4* have been associated with autosomal recessive cutis laxa type 1C,^40–42^ Duchenne Muscular Dystrophy (DMD),^43^ fibrosis-related disorders,^44^ cancer,^45^ pulmonary disorders, and cardiovascular disorders.^46^ PedSep-D patients had the most severe kidney involvement, whereas *LTBP4* was found to protect against tubular interstitial fibrosis by strengthening angiogenesis, downregulating inflammatory gene expression, and facilitating the maintenance of mitochondrial structure in tubular epithelial cells.^47^ Common variants in *LTBP4* have previously been reported in GWASs to be associated with several traits, including lung function (FEV1/FVC),^48^ peak expiratory flow,^49^ hematocrit and hemoglobin,^50^ eosinophil counts,^51^ carotid intima-media thickness,^52^ and diastolic blood pressure.^53^ The precise molecular mechanism by which deleterious *LTBP4* alleles may contribute to the PedSep-D phenotype awaits future functional studies. It remains to be determined whether known activities of LTBP4, elastic fiber organization and regulation transforming growth factor β (*TGF-β*) signaling^54^ or as yet undiscovered functions play a role in sepsis pathogenesis. *TGF-β* remains an attractive candidate given its potent anti-inflammatory effects.^55^ However, our finding that ADAMTS13 levels mediate part of the LTBP4 effect provide evidence for previously unknown molecular interactions between these proteins in sepsis. Because another ADAMTS protease family member, ADAMTS7 is known to cleave LTBP4,^56^ LTBP4 may serve either as a substrate or a competitive inhibitor of ADAMTS13. In the field of sepsis and trauma, Bergmann et al. have postulated the role of *TGF-β* and connected it, as well as other immunosuppressive cytokines, with the high mortality rate of patients discharged from ICU.^55^

As one of the two genes with suggestive significance, the *PLA2G4E* gene encodes a member of the cytosolic phospholipase A2 group IV family involved in membrane tubule-mediated transport regulation. It plays an important role in trafficking through the clathrin-independent endocytic pathway.^57^ *PLA2G4E* was also up-regulated in Alzheimer’s disease APP-PS1 transgenic mice lacking CD8 T cells compared to the control group.^58^ Common variants in *PLA2G4E* have been reported in previous GWASs to be associated with several sepsis clinical prognostic factors, such as neutrophil count,^59,60^ white blood cell count,^61^ and mean platelet volume.^51^

As another gene displaying suggestive significance, *CCDC157* encodes a protein coiled-coil domain containing 157. Common variants in *CCDC157* have been reported in previous GWASs to be associated with sepsis risk factors, such as hematocrit,^50^ pulse pressure,^62^ and calcium levels.^59^ No clear function of immune dysregulation has been reported for *CCDC157* to date.

Although none of the pathways are significantly associated with PedSep-D phenotype, top-ranked pathways involve important biological processes related to sepsis development. For example, the high rank of N-acylphosphatidylethanolamine metabolic process supported previous investigations revealing evaluated fatty acids as candidate biomarkers of sepsis.^63^ The high rank of growth hormone (GH) secretion was supported by previous studies suggesting GH level is higher in septic shock patients compared to sepsis patients and is also higher in sepsis non-survivors.^64^ Other top pathways involve endocytic recycling that plays a role in infection-host interaction^65^ and regulation of cilium beat frequency which is related to respiratory disease and airway infection.^66^

There are several limitations in this study. First, the tested sample size is small, limiting the statistical power to detect associations. As such, larger independent cohorts are needed for validation and meta-analysis. Second, the signals from rare variants may be caused by local ancestry differences, in which situation the number of alleles derived from distinct ancestral populations at a given locus is different. Therefore, although we account for global ancestry by adjusting for top PCs in the association test and conducting sensitivity analysis, it is crucial to further perform local ancestry inference and examine the results in diverse populations separately to validate our findings. Third, we acknowledge the potential impact of residual LD on our association findings. Although we implemented LD pruning, it is challenging to fully resolve LD structure, particularly in regions with complex haplotypes or in diverse populations where reference panels may be limited. As such, some observed associations may reflect correlated signals rather than direct causal effects. Fourth, while our current analysis focused on individual variant and gene-level associations, future studies leveraging polygenic risk scores or burden scores that integrate both common and rare variants may offer a more continuous and potentially powerful framework for assessing genetic contributions to disease severity and subtype classification in sepsis. For example, Rautanen et al. identified genetic variants associated with 28-day survival in sepsis, providing a foundation for polygenic modeling of sepsis outcomes.^12^ The application of PRS in this context is an emerging area and will benefit from larger datasets and phenotype-specific GWAS to enable accurate score derivation. As a secondary analysis, we examined GTEx-based tissue-specific expression profiles to provide biological context for the potential regulatory function of the identified variants, particularly in tissues implicated in sepsis pathophysiology. However, we recognize that GTEx may not fully reflect gene expression dynamics in children with sepsis. To address this limitation and strengthen the biological relevance of our findings, future studies incorporating transcriptomic or proteomic data from pediatric sepsis patients and age-matched healthy controls will be essential for validation. Finally, given the nonsignificant findings from the GAUSS method, interpretation of the pathway analysis results should be approached with caution, and the results require further validation in a larger cohort. In summary, our study identified rare variants that, if found to have functional effects in future studies, might play a role in pediatric sepsis outcomes, providing evidence for a genetic contribution to disease heterogeneity.

## Supplementary Material

Supplemental Files

**Supplementary information** The online version contains supplementary material available at https://doi.org/10.1038/s41390-025-04420-3.

## Figures and Tables

**Table 1.** GAUSS pathway-based association test results.

Pathway term	p-value	Core set
N acylphosphatidylethanolamine metabolic process	0.0011	PLA2G4E
Growth hormone secretion	0.003	LTBP4
Positive regulation of endocytic recycling	0.003	PLA2G4E
Regulation of cilium beat frequency	0.01	MKKS, CATSPER1, CCDC39, BBS2, GAS2L2, DNAH11
Regulation of endocytic recycling	0.013	PLA2G4E
Regulation of cilium beat frequency involved in ciliary motility	0.015	MKKS, CATSPER1, BBS2, GAS2L2
Response to phenylpropanoid	0.015	UGT3A2, EGFR
Negative regulation of cell adhesion molecule production	0.019	NOTCH1, MYOCD, NOTCH4
Histone threonine phosphorylation	0.023	PKN1
Regulation of artery morphogenesis	0.025	NOTCH1
Regulation of lymphoid progenitor cell differentiation	0.026	ZBTB1, NOTCH1
Neuron neuron synaptic transmission	0.029	KIF1B, DLGAP2
Positive regulation of fear response	0.029	PENK
Epoxide metabolic process	0.032	EPHX1
Coronary artery morphogenesis	0.038	NOTCH1, SEC24B, LRP2
Meiotic chromosome condensation	0.039	NCAPD2
Smoothened signaling pathway involved in regulation of cerebellar granule cell precursor cell proliferation	0.039	GLI2, ZNF423
Kinetochore assembly	0.039	CENPT
Dentinogenesis	0.046	TCIRG1, SLC34A1
Aggressive behavior	0.049	PENK

**Table 2.** Demographic and day one clinical characteristics of PedSep-D and Non-PedSep-D patients.

Characteristics	PedSep-D	Non-PedSep-D	p-value^a^
**No. of Patients, *N*** (%)	40 (12.539)	279 (87.461)	
**Demographic**			
Age, years mean (SD)	8 (6)	6 (6)	0.242
Male, N (%)	25 (62.5)	150 (53.8)	0.385
Hispanic, N (%)	3 (7.7)	47 (17.6)	0.278
Previous healthy, N (%)	17 (42.5)	136 (48.7)	0.569
Surgery, N (%)	8 (20.0)	30 (10.8)	0.153
**Organ Dysfunction**			
SIRS criteria^b^, mean (SD)	3.0 (0.8)	2.9 (0.8)	0.477
OFI^c^, mean (SD)	3.0 (1.1)	1.6 (0.6)	<0.001
**Inflammation**			
CRPH, mg/dL mean (SD)	12.8 (11.6)	11.5 (9.9)	0.744
Low temperature, °C mean (SD)	36.4 (1.0)	36.6 (1.3)	0.190
High temperature, °C mean (SD)	37.9 (1.4)	37.8 (1.2)	0.890
ALC, /mm^3^ median (IQR)	1.3 (0.7–2.4)	1.3 (0.7–2.2)	0.685
Ferritin, ng/mL mean (IQR)	575.0 (195.6–1628.8)	180.0 (87.4–403.0)	<0.001
**Pulmonary**			
Pulmonary OFI, N (%)	25 (62.5)	188 (67.4)	0.665
Intubation, N (%)	22 (55.0)	156 (55.9)	1.000
**Cardiovascular or Hemodynamic**			
Heart rate, bpm mean (SD)	145.5 (38.8)	156.3 (30.6)	0.095
Systolic blood pressure, mmHg mean (SD)	79.1 (22.2)	81.7 (19.3)	0.298
CV OFI, N (%)	30 (75.0)	189 (67.7)	0.457
**Renal**			
Creatinine, mg/dL median (IQR)	1.5 (1.0–3.0)	0.4 (0.3–0.7)	<0.001
Renal OFI, N (%)	26 (65.0)	0 (0.0)	<0.001
**Hepatic**			
Hepatic OFI, N (%)	12 (30.0)	19 (6.8)	<0.001
**Hematologic**			
Hemoglobin, g/dL mean (SD)	9.4 (1.8)	10.0 (1.9)	0.097
Platelets, K/mm^3^ mean (SD)	84.0 (73.7)	192.9 (112.9)	<0.001
Hematologic OFI, N (%)	19 (47.5)	7 (2.5)	<0.001
**Other**			
Glasgow Coma Scale score^d,e^, mean (SD)	7.5 (5.6)	8.5 (5.3)	0.400
CNS OFI, N (%)	9 (22.5)	33 (11.8)	0.106
**Comorbid Conditions**			
Leukemia, N (%)	2 (5.0)	9 (3.2)	0.635
Hemolytic Anemia, N (%)	1 (2.5)	1 (0.4)	0.235
Rheumatic Disease, N (%)	2 (5.0)	5 (1.8)	0.215
IBD, N (%)	2 (5.0)	0 (0)	0.015
Renal Disease, N (%)	2 (5.0)	3 (1.1)	0.120
Chromosome Abnormal, N (%)	9 (22.5)	37 (13.3)	0.189
Metabolic Disease, N (%)	0 (0)	10 (3.6)	0.620
Diabetes, N (%)	1 (2.5)	2 (0.7)	0.332
Cardiovascular Disease, N (%)	7 (17.5)	43 (15.4)	0.915
Trauma, N (%)	0 (0)	4 (1.4)	1.000
Short Gut, N (%)	0 (0)	7 (2.5)	0.602
Liver Disease, N (%)	2 (5.0)	7 (2.5)	0.314

*IQR* interquartile range, *SIRS* systemic inflammatory response syndrome, *OFI* organ failure index, *CRPH* high-sensitivity cardiac C-Reactive protein, *ALC* absolute lymphocyte count, *CNS* central nervous system.

SI conversion factors: to convert alanine transaminase and aspartate aminotransferase to μkat/L, multiply by 0.0167; bilirubin to μmol/L, multiply by 17.104; C-reactive protein to nmol/L, multiply by 9.524; creatinine to μmol/L, multiply by 88.4.

a Comparisons across all four phenotypes were performed using the Kruskal–Wallis test, the χ2 test, or Fisher’s exact test.

b Indicates SIRS criteria ranging from 0 to 4, including abnormal heart rate, respiratory rate, temperature, and white blood cell count.

c OFI is an integer score reflecting the number of organ failures. Scores are either 0 or 1 for cardiovascular, hepatic, hematologic, respiratory, neurological, and renal, and summed for a total range of 0 to 6. Cardiovascular, need for cardiovascular agent infusion support; Pulmonary, need for mechanical ventilation support with the ratio of the arterial partial pressure of oxygen and the fraction of inspired oxygen (PaO2/FiO2) < 300 without this support; Hepatic, total bilirubin > 1.0 mg/dL and alanine aminotransferase (ALT) > 100 units/L; Renal, serum creatinine > 1.0 mg/dL and oliguria (urine output < 0.5 mL/kg/h); Hematologic, thrombocytopenia < 100,000/mm3 and prothrombin time INR > 1.5 × normal; Central Nervous System, Glasgow Coma Scale (GCS) Score < 12 in the absence of sedatives.

d Corresponds to the minimum or maximum value (as appropriate) within six h of hospital presentation

e GCS ranges from 3 to 15.

**Table 3.** SKAT gene-based association test result for PedSep-D.

Gene	Chr	# carriers in case (%)	# carriers in control (%)	Odds Ratio	P-value
LTBP4	19	6(15)	2 (0.7)	24.4	1.069e-05
PLA2G4E	15	4(10)	2 (0.7)	15.4	3.288e-05
CCDC157	22	7 (17.5)	10 (3.6)	5.7	6.192e-05

**Table 4.** Characteristics of rare variant carriers and non-carriers.

characteristic	LTBP4		PLA2G4E		CCDC157	
	carriers	Non-carriers	carriers	Non-carriers	carriers	Non-carriers
No. of Patients	8	311	6	313	17	302
Age, median (IQR), y	8 (5, 12)	5 (1, 12)	1 (1, 2)	5 (1, 12)^a^	3 (2, 10)	5 (1, 12)
Sex, N (%)						
Female	3 (37.5)	141 (45.3)	4 (66.7)	140 (44.7)	7 (41.1)	137 (45.4)
Male	5 (62.5)	170 (54.7)	2 (33.3)	173 (55.3)	10 (58.9)	165 (54.6)
Race, N (%)						
White	3 (37.0)	210 (67.5)	5 (83.3)	208 (66.5)	12 (70.6)	201 (66.6)
Black	4 (50.0)	63 (20.3)	1 (16.7)	67 (21.4)	4 (23.5)	63 (20.9)
Asian	0 (0.0)	14 (4.5)	0 (0.0)	14 (4.5)	1 (5.9)	14 (4.6)
Other	1 (13.0)	24 (7.7)	0 (0.0)	24 (7.7)	0 (0.0)	24 (7.9)
Ethnicity, N (%)						
Non-Hispanic	6 (75.0)	250 (80.4)	6 (100.0)	250 (79.9)	13 (76.5)	243 (80.5)
Hispanic	2 (25.0)	48 (15.4)	0 (0.0)	50 (16.0)	2 (11.8)	48 (15.9)
Unknown	0 (0.0)	13 (4.2)	0 (0.0)	13 (4.2)	2 (11.8)	11 (3.6)
Previous healthy	5 (62.5)	148 (47.6)	3 (50.0)	150 (47.9)	8 (47.1)	145 (48.0)
Immunocompromised, N (%)	1 (12.5)	58 (18.6)	1 (16.7)	58 (18.5)	3 (17.6)	56 (18.5)
PRISM Score, median (IQR)	8.5 (7.25, 15.75)	8 (3, 15)	18.5 (15.75, 19.00)^a^	8 (3, 14)	10 (3, 15)	8 (3, 15)
OFI, median (IQR)	2.5 (2, 3)^a^	2 (1, 2)	2 (1, 3.75)	2 (1, 2)	2 (2, 3)^a^	2 (1, 2)
Infection, N (%)						
Bacterial infection	1 (12.5)	113 (36.3)	1 (16.7)	113 (36.1)	7 (41.1)	107 (35.4)
Viral infection	0 (0.0)	88 (28.3)	2 (33.3)	86 (27.5)	2 (11.8)	86 (28.5)
Fungal infection	0 (0.0)	2 (0.6)	0 (0.0)	2 (0.6)	0 (0.0)	2 (0.7)
No infection	7 (87.5)	108 (34.7)	3 (50.0)	110 (35.1)	8 (47.1)	107 (35.4)
Mortality, N (%)	2 (25.0)	26 (8.4)	1 (16.7)	27 (8.6)	1 (5.9)	27 (8.9)

a The value in this group is significantly higher than the compared group.

**Table 5.** Single variant association and functional prediction for variants contributing to the gene-level significance.

Gene	Variant	SNP Information^a^	Amino acid change^b^	CADD score^c^	GERP score^d^	SIFT score^e^	Polyphen2 score^f^
LTBP4	rs370696272	19:41105311:C:T	Leu27Leu	17.55	1.63	-	-
	rs573310430	19:41122842:C:T	Arg984Cys	34	4.63	0.044 (D)	1.0 (D)
	-	19:41132970:C:T	Pro1388Leu	25.8	4.58	0.68 (T)	0.998 (D)
	rs200607327	19:41133005:G:A	Gly1437Arg	27	4.58	0.38 (T)	1.0 (D)
PLA2G4E	-	15:42276733:T:G	Lys387Gln	23	4.48	0.275 (T)	0.26 (B)
	rs764494895	15:42278161:G:A	Ala693Val	11.04	0.591	0.25 (T)	0.004 (B)
	rs143966595	15:42293394:C:T	Val212Ile	23.1	5.34	0.099 (T)	0.05 (B)
	rs776016335	15:42298270:T:C	Asp148Gly	27.1	5.66	0.002 (D)	1.0 (D)
CCDC157	rs9606721	22:30762035:A:G	Thr16Ala	12.47	1.76	0.28 (T)	0.001 (B)
	rs540507025	22:30762080:C:T	Arg31Cys	23.1	2.89	0.002 (D)	1.0 (D)
	rs143249037	22:30766366:G:A	Glu158Lys	14.06	1.36	0.282 (T)	0.035 (B)
	-	22:30766438:C:A	Gln182Lys	8.248	2.89	0.931 (T)	0.009 (B)
	rs139609945	22:30766496:C:T	Thr201Met	8.755	2.19	0.107 (T)	0.155 (B)
	rs1235664314	22:30766672:G:T	Asp260Tyr	28.4	5.29	0.008 (D)	1.0 (D)
	rs148283823	22:30766868:G:A	Arg325Gln	6.266	0.566	0.712 (T)	0.093 (B)
	rs202178544	22:30772567:T:C	Ser698Pro	0.246	−2.83	0.339 (T)	0.0 (B)

a SNPs are listed as chromosome: position (hg19): reference allele: alternative allele.

b Amino acid substitutions caused by SNPs.

c CADD (Combined Annotation-Dependent Depletion) score measures the predicted variant effect rank, higher value implies a greater damaging effect throughout the human genome reference assembly. A score of 10 indicates that the SNP is predicted to be in the top 10% most deleterious substitutions in the human genome, a score 0f 20 indicates that the SNP is predicted to be in the top 1% most deleterious substitutions, a score of 30 indicates that the SNP is predicted to be in the top 0.1% most deleterious substitutions and so forth.

d GERP (Genomic Evolutionary Rate Profiling) score indicates position-specific estimates of evolutionary constraint. A positive score scale with the level of constraint, a greater score suggests a greater level of evolutionary constraint. A negative score indicates that a site is probably evolving neutrally.

e SIFT (Sorting Intolerant from tolerant) score ranges from 0 to 1. A value less than 0.05 is classified as damaging (D), whereas a higher score is classified as tolerated (T).

f Polyphen2 (Polymorphism Phenotyping v2) score ranges from 0 to 1. Value implies probably damaging” (“D”) for scores in [0.957, 1]; “possibly damaging” (“D”) for scores in [0.453, 0.956]; “benign” (“B”) for scores in [0, 0.452].

## Data Availability

Sepsis exome data is available upon request. However, as the data is governed by IRB regulations, requesters must ensure that the IRB policies at their institution are compatible with the regulations under which the data is protected.

## IMPACT:

- Pediatric sepsis exhibits substantial heterogeneity, with genetic variation contributing to this variability. Rare variants in LTBP4 are significantly associated with the most severe pediatric sepsis phenotype (PedSep-D), while variants in PLA2G4E and CCDC157 show associations with this phenotype in suggestive significance.
- Expands on the concept of sepsis phenotypes (PedSep-A, B, C, D) by incorporating genetic insights, moving beyond clinical and cytokine profiles to uncover molecular drivers.
- Opens new avenues for mechanistic studies to understand the genetic underpinnings of severe inflammation and immune activation in sepsis.
