# Supplementary figures and images for: Deleterious variants in LTBP4 are associated with severe pediatric sepsis
Source: Pediatr Res. 2025 Oct 11;99(5):2007–18. doi: 10.1038/s41390-025-04420-3 (PMC13182162; doi:10.1038/s41390-025-04420-3)

**S. Figure 1. Manhattan plot for PedSep-A (No. of genes = 3,846)**


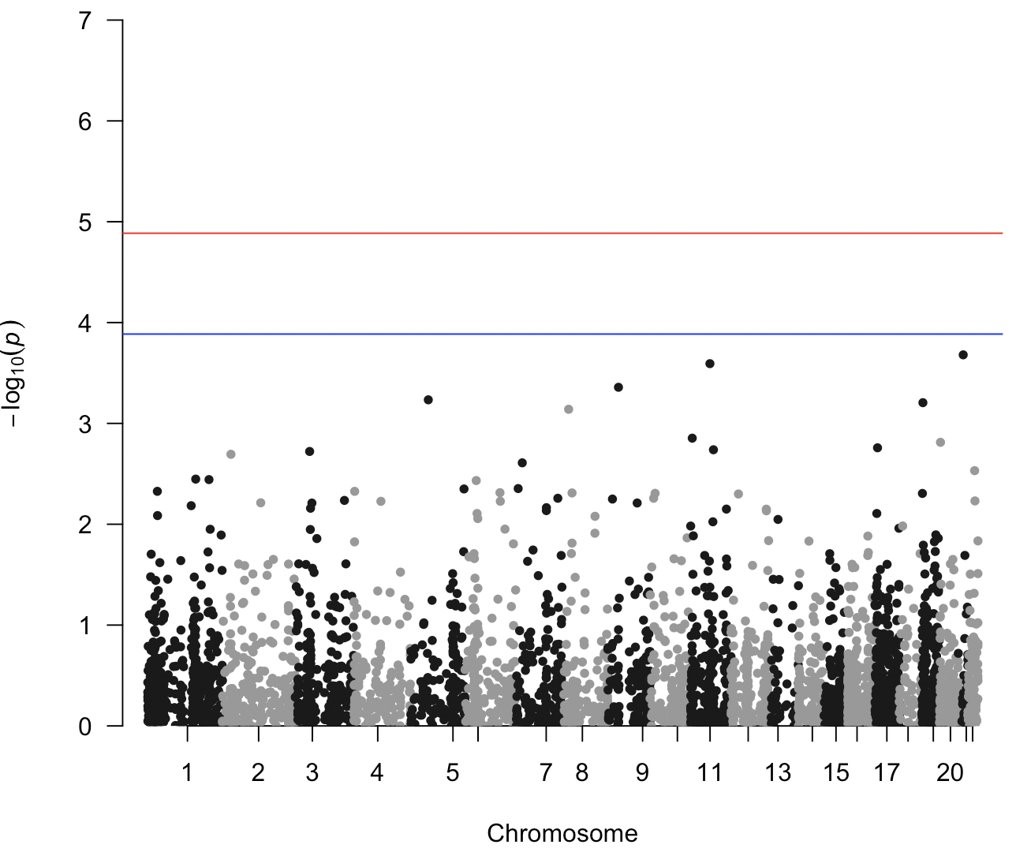

Supplement: Supplementary file 1 — S. Figure 1 [file 41390_2025_4420_MOESM1_ESM.docx]

**S. Figure 2. Manhattan plot for PedSep-B (No. of genes = 3,846)**


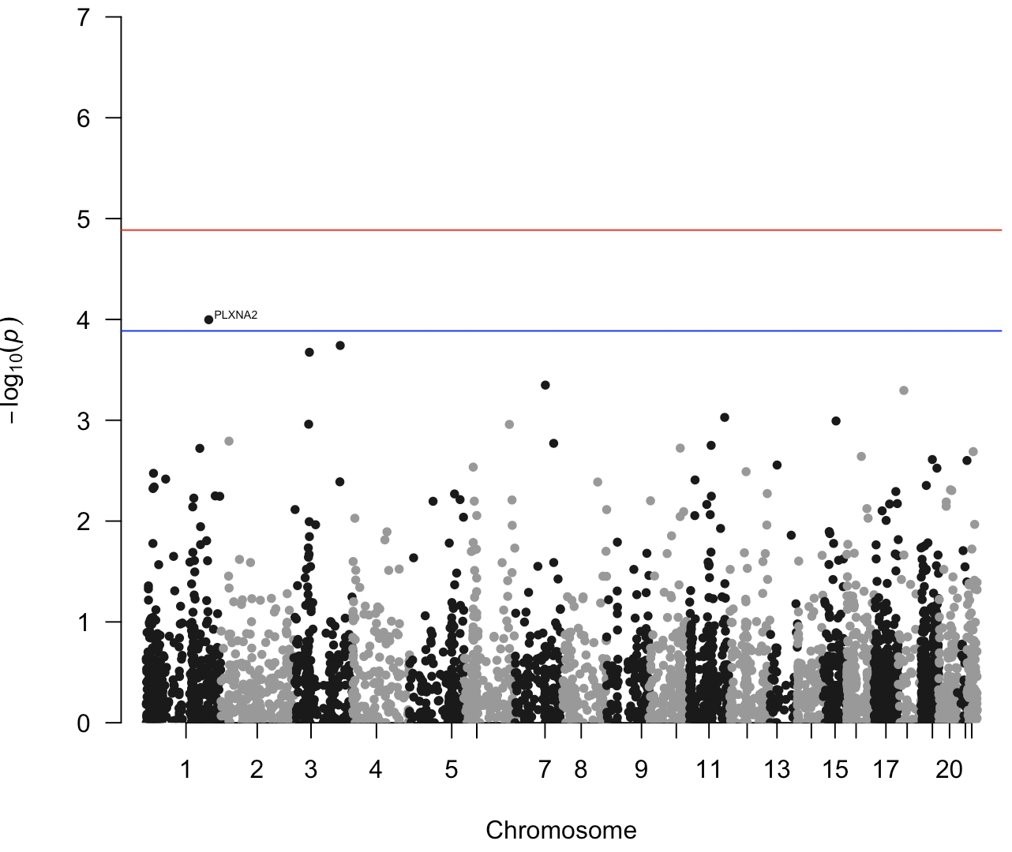

Supplement: Supplementary file 2 — S. Figure 2 [file 41390_2025_4420_MOESM2_ESM.docx]

**S. Figure 3. Manhattan plot for PedSep-C (No. of genes = 3,846)**


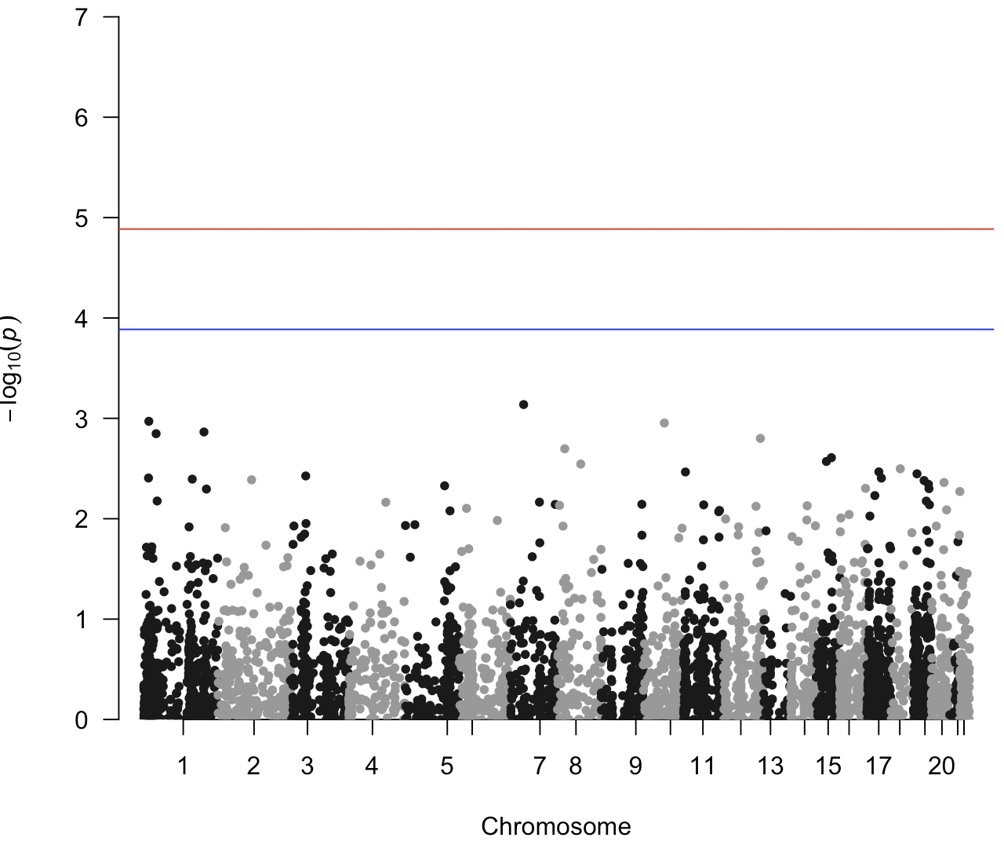

Supplement: Supplementary file 3 — S. Figure 3 [file 41390_2025_4420_MOESM3_ESM.docx]

**S. Figure 4. QQ plot of four phenotypes**


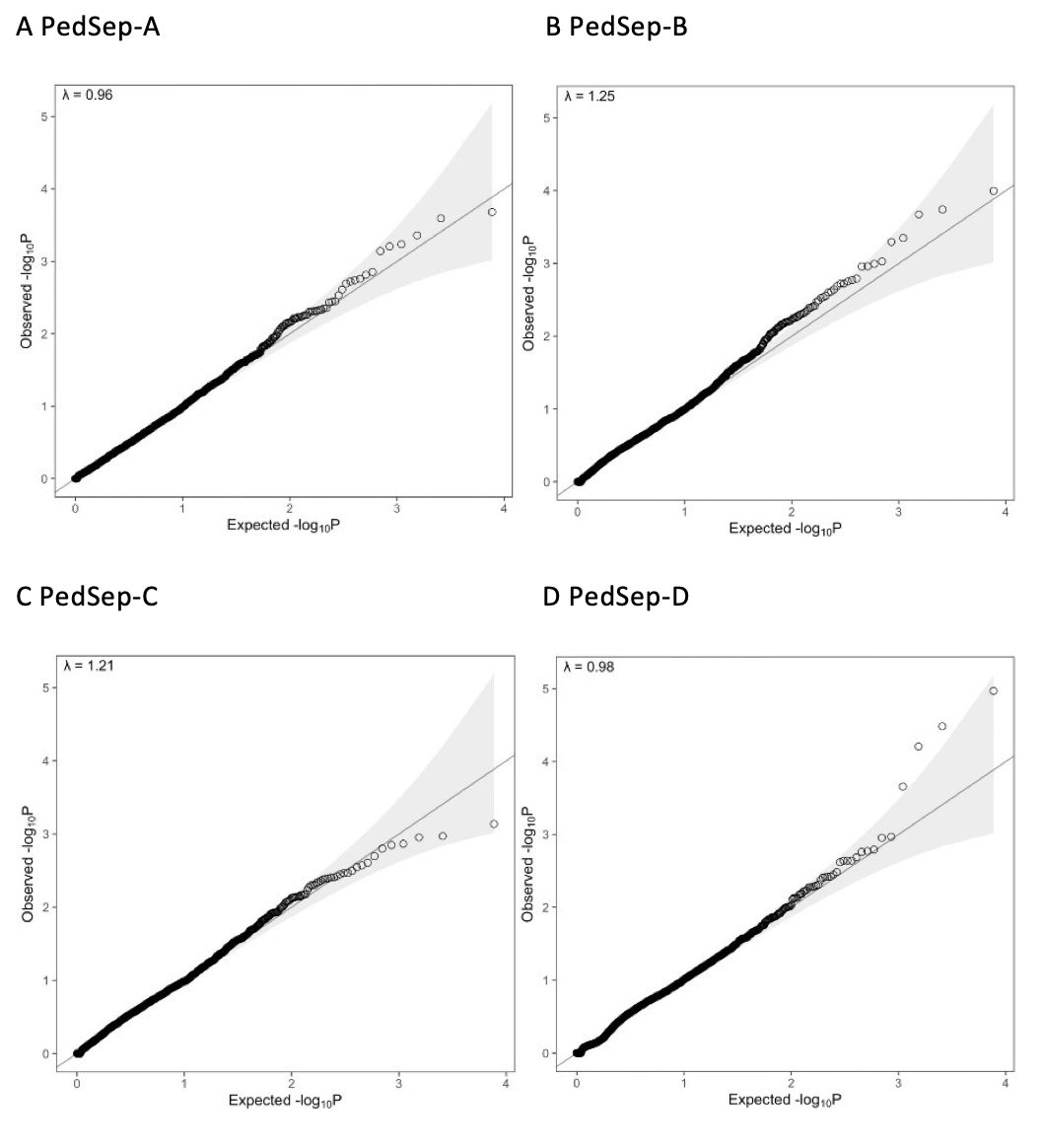

Supplement: Supplementary file 4 — S. Figure 4 [file 41390_2025_4420_MOESM4_ESM.docx]
