# Supplementary material for: Deleterious variants in LTBP4 are associated with severe pediatric sepsis
Source: Pediatr Res. 2025 Oct 11;99(5):2007–18. doi: 10.1038/s41390-025-04420-3 (PMC13182162; doi:10.1038/s41390-025-04420-3)
Supplement: Supplementary file 5 — S. Table 1 [file 41390_2025_4420_MOESM5_ESM.docx]

**S. Table 1. Demographic and day 1 clinical characteristics of PedSep-A and Non-PedSep-A patients**

| **Characteristics** | **PedSep-A** | **Non-PedSep-A** | **p-value^1^** |
| --- | --- | --- | --- |
| **No. of Patients, *N* (%)** | 116 (36.4) | 203 (63.6) |  |
| **Demographic** |  |  |  |
| Age, years mean (SD) | 3 (4) | 9 (6) | <0.001 |
| Male, N (%) | 52 (44.8) | 123 (60.6) | 0.009 |
| Hispanic, N (%) | 24 (22) | 26 (13.2) | 0.056 |
| Previous healthy, N (%) | 79 (68.1) | 74 (36.5) | <0.001 |
| Surgery, N (%) | 3 (2.6) | 35 (17.2) | <0.001 |
| **Organ Dysfunction** |  |  |  |
| SIRS criteria^2^, mean (SD) | 2.9 (0.8) | 2.9 (0.8) | 0.900 |
| OFI^3^, mean (SD) | 1.3 (0.5) | 2.0 (0.9) | <0.001 |
| **Inflammation** |  |  |  |
| CRPH, mg/dL mean (SD) | 7.4 (7.3) | 14.1 (10.7) | <0.001 |
| Low temperature, °C mean (SD) | 36.7 (0.9) | 36.5 (1.4) | 0.639 |
| High temperature, °C mean (SD) | 37.8 (1.0) | 37.8 (1.4) | 0.912 |
| ALC, /mm^3^ median (IQR) | 1.9 (1.3-3.4) | 1.0 (0.5-1.7) | <0.001 |
| Ferritin, ng/mL mean (IQR) | 204.2 (71.5-210.5) | 260.7 (130.7-681.6) | <0.001 |
| **Pulmonary** |  |  |  |
| Pulmonary OFI, N (%) | 89 (76.7) | 124 (61.1) | 0.006 |
| Intubation, N (%) | 62 (53.4) | 116 (57.1) | 0.602 |
| **Cardiovascular or Hemodynamic** |  |  |  |
| Heart rate, bpm mean (SD) | 168.6 (29.6) | 147.1 (30.5) | <0.001 |
| Systolic blood pressure, mmHg mean (SD) | 85.1 (15.9) | 79.2 (21.3) | 0.007 |
| CV OFI, N (%) | 54 (46.6) | 165 (81.3) | <0.001 |
| **Renal** |  |  |  |
| Creatinine, mg/dL median (IQR) | 0.3 (0.2-0.4) | 0.6 (0.4-1.2) | <0.001 |
| Renal OFI, N (%) | 0 (0.0) | 26 (12.8) | <0.001 |
| **Hepatic** |  |  |  |
| Hepatic OFI, N (%) | 3 (2.6) | 28 (13.8) | <0.001 |
| **Hematologic** |  |  |  |
| Hemoglobin, g/dL mean (SD) | 10.1 (1.8) | 9.8 (2.0) | 0.253 |
| Platelets, K/mm^3^ mean (SD) | 257.2 (110.3) | 134.7 (90.7) | <0.001 |
| Hematologic OFI, N (%) | 0 (0.0) | 26 (12.8) | <0.001 |
| **Other** |  |  |  |
| Glasgow Coma Scale score^4,5^, mean (SD) | 8.4 (5.2) | 8.3 (5.4) | 0.840 |
| CNS OFI, N (%) | 10 (8.6) | 32 (15.8) | 0.100 |

IQR interquartile range, SIRS systemic inflammatory response syndrome, OFI organ failure index, ALC absolute lymphocyte count, CNS central nervous system

SI conversion factors: to convert alanine transaminase and aspartate aminotransferase to μkat/L, multiply by 0.0167; bilirubin to μmol/L, multiply by 17.104; C-reactive protein to nmol/L, multiply by 9.524; creatinine to μmol/L, multiply by 88.4

1 Comparisons across all 4 phenotypes were performed using the Kruskal–Wallis test, the χ2 test, or the Fisher’s exact test

2 Indicates SIRS criteria ranging from 0 to 4 including abnormal heart rate, respiratory rate, temperature, and white blood cell count

3 OFI is an integer score reflecting the number of organ failures. Scores are either 0 or 1 for cardiovascular, hepatic, hematologic, respiratory, neurological, and renal, and summed for total range of 0 to 6. Cardiovascular, need for cardiovascular agent infusion support; Pulmonary, need for mechanical ventilation support with the ratio of the arterial partial pressure of oxygen and the fraction of inspired oxygen (PaO2/FiO2) < 300 without this support; Hepatic, total bilirubin > 1.0 mg/dL and alanine aminotransferase (ALT) > 100 units/L; Renal, serum creatinine > 1.0 mg/dL and oliguria (urine output < 0.5 mL/kg/h); Hematologic, thrombocytopenia < 100,000/mm3 and prothrombin time INR > 1.5 × normal; Central Nervous System, Glasgow Coma Scale (GCS) Score < 12 in the absence of sedatives

4 Corresponds to minimum or maximum value (as appropriate) within 6 h of hospital presentation

5 GCS ranges from 3 to 15
