# Supplementary material for: Deleterious variants in LTBP4 are associated with severe pediatric sepsis
Source: Pediatr Res. 2025 Oct 11;99(5):2007–18. doi: 10.1038/s41390-025-04420-3 (PMC13182162; doi:10.1038/s41390-025-04420-3)
Supplement: Supplementary file 6 — S. Table 2 [file 41390_2025_4420_MOESM6_ESM.docx]

**S. Table 2. Demographic and day 1 clinical characteristics of PedSep-B and Non-PedSep-B patients**

| **Characteristics** | **PedSep-B** | **Non-PedSep-B** | **p-value^1^** |
| --- | --- | --- | --- |
| **No. of Patients, *N* (%)** | 86 (26.959) | 233 (73.041) |  |
| **Demographic** |  |  |  |
| Age, years mean (SD) | 8 (6) | 6 (6) | 0.064 |
| Male, N (%) | 56 (65.1) | 119 (51.1) | 0.035 |
| Hispanic, N (%) | 10 (12.2) | 40 (17.9) | 0.464 |
| Previous healthy, N (%) | 26 (30.2) | 127 (54.5) | <0.001 |
| Surgery, N (%) | 18 (20.9) | 20 (8.6) | 0.005 |
| **Organ Dysfunction** |  |  |  |
| SIRS criteria^2^, mean (SD) | 3.0 (0.8) | 2.9 (0.8) | 0.401 |
| OFI^3^, mean (SD) | 2.1 (0.6) | 1.6 (0.9) | <0.001 |
| **Inflammation** |  |  |  |
| CRPH, mg/dL mean (SD) | 13.5 (11.1) | 11.0 (9.6) | 0.064 |
| Low temperature, °C mean (SD) | 35.9 (1.7) | 36.8 (0.9) | <0.001 |
| High temperature, °C mean (SD) | 37.3 (1.3) | 38.0 (1.2) | <0.001 |
| ALC, /mm^3^ median (IQR) | 1.1 (0.7-2.0) | 1.3 (0.7-2.4) | 0.395 |
| Ferritin, ng/mL mean (IQR) | 198.2 (111.6-535.2) | 183.0 (89.0-481.7) | 0.360 |
| **Pulmonary** |  |  |  |
| Pulmonary OFI, N (%) | 73 (84.9) | 140 (60.1) | <0.001 |
| Intubation, N (%) | 80 (93.0) | 98 (42.1) | <0.001 |
| **Cardiovascular or Hemodynamic** |  |  |  |
| Heart rate, bpm mean (SD) | 144.9 (29.4) | 158.6 (32.0) | <0.001 |
| Systolic blood pressure, mmHg mean (SD) | 73.5 (22.0) | 84.3 (17.9) | <0.001 |
| CV OFI, N (%) | 78 (90.7) | 141 (60.5) | <0.001 |
| **Renal** |  |  |  |
| Creatinine, mg/dL median (IQR) | 0.6 (0.3-0.8) | 0.4 (0.3-0.8) | 0.025 |
| Renal OFI, N (%) | 0 (0.0) | 26 (11.2) | <0.001 |
| **Hepatic** |  |  |  |
| Hepatic OFI, N (%) | 9 (10.5) | 22 (9.4) | 0.832 |
| **Hematologic** |  |  |  |
| Hemoglobin, g/dL mean (SD) | 9.6 (2.1) | 10.1 (1.9) | 0.031 |
| Platelets, K/mm^3^ mean (SD) | 155.7 (96.2) | 187.9 (119.5) | 0.033 |
| Hematologic OFI, N (%) | 0 (0.0) | 26 (11.2) | <0.001 |
| **Other** |  |  |  |
| Glasgow Coma Scale score^4,5^, mean (SD) | 4.5 (3.3) | 9.8 (5.2) | <0.001 |
| CNS OFI, N (%) | 19 (22.1) | 23 (9.9) | 0.007 |

IQR interquartile range, SIRS systemic inflammatory response syndrome, OFI organ failure index, ALC absolute lymphocyte count, CNS central nervous system

SI conversion factors: to convert alanine transaminase and aspartate aminotransferase to μkat/L, multiply by 0.0167; bilirubin to μmol/L, multiply by 17.104; C-reactive protein to nmol/L, multiply by 9.524; creatinine to μmol/L, multiply by 88.4

1 Comparisons across all 4 phenotypes were performed using the Kruskal–Wallis test, the χ2 test, or the Fisher’s exact test

2 Indicates SIRS criteria ranging from 0 to 4 including abnormal heart rate, respiratory rate, temperature, and white blood cell count

3 OFI is an integer score reflecting the number of organ failures. Scores are either 0 or 1 for cardiovascular, hepatic, hematologic, respiratory, neurological, and renal, and summed for total range of 0 to 6. Cardiovascular, need for cardiovascular agent infusion support; Pulmonary, need for mechanical ventilation support with the ratio of the arterial partial pressure of oxygen and the fraction of inspired oxygen (PaO2/FiO2) < 300 without this support; Hepatic, total bilirubin > 1.0 mg/dL and alanine aminotransferase (ALT) > 100 units/L; Renal, serum creatinine > 1.0 mg/dL and oliguria (urine output < 0.5 mL/kg/h); Hematologic, thrombocytopenia < 100,000/mm3 and prothrombin time INR > 1.5 × normal; Central Nervous System, Glasgow Coma Scale (GCS) Score < 12 in the absence of sedatives

4 Corresponds to minimum or maximum value (as appropriate) within 6 h of hospital presentation

5 GCS ranges from 3 to 15
