# Supplementary material for: Deleterious variants in LTBP4 are associated with severe pediatric sepsis
Source: Pediatr Res. 2025 Oct 11;99(5):2007–18. doi: 10.1038/s41390-025-04420-3 (PMC13182162; doi:10.1038/s41390-025-04420-3)
Supplement: Supplementary file 7 — S. Table 3 [file 41390_2025_4420_MOESM7_ESM.docx]

**S. Table 3. Demographic and day 1 clinical characteristics of PedSep-C and Non-PedSep-C patients**

| **Characteristics** | **PedSep-C** | **Non-PedSep-C** | **p-value^1^** |
| --- | --- | --- | --- |
| **No. of Patients, *N* (%)** | 77 (24.138) | 242 (75.862) |  |
| **Demographic** |  |  |  |
| Age, years mean (SD) | 10 (5) | 6 (6) | <0.001 |
| Male, N (%) | 42 (54.5) | 133 (55.0) | 1.000 |
| Hispanic, N (%) | 13 (17.1) | 37 (16.1) | 0.409 |
| Previous healthy, N (%) | 31 (40.3) | 122.0 (50.4) | 0.155 |
| Surgery, N (%) | 9 (11.7) | 29 (12.0) | 1.000 |
| **Organ Dysfunction** |  |  |  |
| SIRS criteria^2^, mean (SD) | 2.8 (0.8) | 2.9 (0.8) | 0.200 |
| OFI^3^, mean (SD) | 1.3 (0.5) | 1.9 (0.9) | <0.001 |
| **Inflammation** |  |  |  |
| CRPH, mg/dL mean (SD) | 15.4 (9.6) | 10.5 (10.0) | <0.001 |
| Low temperature, °C mean (SD) | 37.2 (0.9) | 36.4 (1.3) | <0.001 |
| High temperature, °C mean (SD) | 38.4 (1.3) | 37.6 (1.2) | <0.001 |
| ALC, /mm^3^ median (IQR) | 0.6 (0.3-1.1) | 1.5 (1.0-2.7) | <0.001 |
| Ferritin, ng/mL mean (IQR) | 260.7 (165.0-3616.4) | 173.5 (87.1-443.8) | <0.001 |
| **Pulmonary** |  |  |  |
| Pulmonary OFI, N (%) | 26 (33.8) | 187 (77.3) | <0.001 |
| Intubation, N (%) | 14 (18.2) | 164.0 (67.8) | <0.001 |
| **Cardiovascular or Hemodynamic** |  |  |  |
| Heart rate, bpm mean (SD) | 150.4 (26.7) | 156.4 (33.3) | 0.093 |
| Systolic blood pressure, mmHg mean (SD) | 85.7 (18.0) | 80 (20.0) | 0.080 |
| CV OFI, N (%) | 57 (74.0) | 162 (66.9) | 0.305 |
| **Renal** |  |  |  |
| Creatinine, mg/dL median (IQR) | 0.5 (0.4-0.7) | 0.4 (0.3-0.9) | 0.090 |
| Renal OFI, N (%) | 0 (0.0) | 26 (10.7) | 0.001 |
| **Hepatic** |  |  |  |
| Hepatic OFI, N (%) | 7 (9.1) | 24 (9.9) | 1.000 |
| **Hematologic** |  |  |  |
| Hemoglobin, g/dL mean (SD) | 10.4 (1.9) | 9.8 (1.9) | 0.025 |
| Platelets, K/mm^3^ mean (SD) | 137.6 (82.9) | 192.5 (120.0) | <0.001 |
| Hematologic OFI, N (%) | 7 (9.1) | 19 (7.9) | 0.811 |
| **Other** |  |  |  |
| Glasgow Coma Scale score^4,5^, mean (SD) | 13.1 (3.2) | 6.9 (5.0) | <0.001 |
| CNS OFI, N (%) | 4 (5.2) | 38 (15.7) | 0.029 |

IQR interquartile range, SIRS systemic inflammatory response syndrome, OFI organ failure index, ALC absolute lymphocyte count, CNS central nervous system

SI conversion factors: to convert alanine transaminase and aspartate aminotransferase to μkat/L, multiply by 0.0167; bilirubin to μmol/L, multiply by 17.104; C-reactive protein to nmol/L, multiply by 9.524; creatinine to μmol/L, multiply by 88.4

1 Comparisons across all 4 phenotypes were performed using the Kruskal–Wallis test, the χ2 test, or the Fisher’s exact test

2 Indicates SIRS criteria ranging from 0 to 4 including abnormal heart rate, respiratory rate, temperature, and white blood cell count

3 OFI is an integer score reflecting the number of organ failures. Scores are either 0 or 1 for cardiovascular, hepatic, hematologic, respiratory, neurological, and renal, and summed for total range of 0 to 6. Cardiovascular, need for cardiovascular agent infusion support; Pulmonary, need for mechanical ventilation support with the ratio of the arterial partial pressure of oxygen and the fraction of inspired oxygen (PaO2/FiO2) < 300 without this support; Hepatic, total bilirubin > 1.0 mg/dL and alanine aminotransferase (ALT) > 100 units/L; Renal, serum creatinine > 1.0 mg/dL and oliguria (urine output < 0.5 mL/kg/h); Hematologic, thrombocytopenia < 100,000/mm3 and prothrombin time INR > 1.5 × normal; Central Nervous System, Glasgow Coma Scale (GCS) Score < 12 in the absence of sedatives

4 Corresponds to minimum or maximum value (as appropriate) within 6 h of hospital presentation

5 GCS ranges from 3 to 15
