# Supplementary material for: Deleterious variants in LTBP4 are associated with severe pediatric sepsis
Source: Pediatr Res. 2025 Oct 11;99(5):2007–18. doi: 10.1038/s41390-025-04420-3 (PMC13182162; doi:10.1038/s41390-025-04420-3)
Supplement: Supplementary file 8 — S. Table 4 [file 41390_2025_4420_MOESM8_ESM.docx]

**S. Table 4. Biomarkers measured at day 1 by phenotype PedSep-A (N = 319)**

| **Biomarker^a^** | **PedSep-A (N = 116)** | **Non-PedSep-A (N = 203)** | **p-value** |
| --- | --- | --- | --- |
| ADAMTS13, % | 83.0 (65.0, 95.0) | 66.0 (48.0, 83.5) | <0.001 |
| SFasLg, pg/ml | 59.8 (39.1, 87.0) | 40.8 (29.4, 66.3) | <0.001 |
| Ex vivo TNF-α, pg/ml | 691.0 (334.0, 1049.2) | 385.9 (97.1, 944.3) | <0.001 |
| TNF-α, pg/ml | 1049.2 (728.0, 1049.2) | 1049.2 (604.1, 1049.2) | 0.721 |
| sCD163, pg/ml | 212829 (155490, 300033) | 340338 (215555, 580440) | <0.001 |
| IFN-β, pg/ml | 6.4 (6.4, 6.4) | 6.4 (6.4, 8.2) | 0.118 |
| IL-22, pg/ml | 22.4 (17.8, 29.5) | 28.3 (22.1, 38.4) | <0.001 |
| IL-18, pg/ml | 329.6 (222.6, 533.6) | 485.9 (294.6, 882.7) | <0.001 |
| IL-18BP, pg/ml | 9445 (6185, 16011) | 20764 (12054, 33449) | <0.001 |
| MIG/CXCL9, pg/ml | 619.3 (406.0, 1061.2) | 1026.0 (490.6, 2883.8) | <0.001 |
| IL-1β, pg/ml | 2.6 (2.1, 3.1) | 2.8 (2.4, 3.3) | 0.071 |
| IL-4, pg/ml | 4.7 (3.5, 6.3) | 4.7 (3.5, 6.5) | 0.799 |
| IL-6, pg/ml | 6.9 (5.8, 9.7) | 10.9 (6.5, 30.2) | <0.001 |
| IL-8, pg/ml | 38.0 (26.4, 65.5) | 59.5 (36.3, 130.5) | <0.001 |
| IL-10, pg/ml | 19.3 (15.4, 24.6) | 25.0 (18.1, 40.3) | <0.001 |
| IL-13, pg/ml | 3.1 (3.1, 4.3) | 3.1 (3.1, 3.5) | 0.416 |
| IL-17A, pg/ml | 17.4 (15.1, 21.7) | 20.0 (16.5, 25.1) | <0.001 |
| IFN-γ, pg/ml | 2.8 (2.8, 3.0) | 2.8 (2.8, 2.8) | 0.477 |
| IP-10/CXCL10, pg/ml | 492.2 (268.5, 1691.1) | 850.5 (381.0, 2361.3) | 0.008 |
| MCP-1/CCL2, pg/ml | 103.5 (48.3, 190.8) | 178.6 (84.3, 400.5) | <0.001 |
| MIP-1α, pg/ml | 0.6 (0.6, 0.6) | 2.0 (0.6, 10.7) | <0.001 |
| MIP-1β, pg/ml | 42.8 (28.1, 57.2) | 50.7 (34.5, 81.1) | <0.001 |
| MCP-3, pg/ml | 92.4 (92.4, 147.8) | 92.4 (92.4, 166.0) | 0.269 |
| IFN-α2, pg/ml | 125.7 (105.8, 142.3) | 125.7 (105.8, 142.8) | 0.542 |
| IL-1α, pg/ml | 9.4 (9.4, 11.6) | 9.4 (9.4, 16.4) | 0.183 |
| IL-2RA, pg/ml | 343.5 (235.8, 504.6) | 401.8 (243.4, 696.7) | 0.014 |
| IL-3, pg/ml | 612.2 (496.1, 734.6) | 612.2 (529.0, 724.4) | 0.976 |
| IL-16, pg/ml | 544.1 (398.1, 660.5) | 635.5 (453.6, 836.7) | <0.001 |
| M-CSF, pg/ml | 19.7 (13.8, 34.0) | 36.2 (21.6, 78.6) | <0.001 |
| SCF, pg/ml | 138.2 (111.4, 192.7) | 167.5 (115.4, 269.8) | <0.001 |
| TRAIL, pg/ml | 42.9 (32.9, 64.2) | 35.4 (27.9, 48.5) | <0.001 |
| CRPH, mg/dL | 4.6 (1.3, 12.4) | 11.4 (6.2, 20.8) | <0.001 |
| Ferritin, ng/mL | 121.0 (71.0, 204.5) | 260.9 (130.6, 682.4) | <0.001 |

^a^ All biomarkers are measured one time concomitantly in the first day. Values in table are summarized as median (IQR)
