# Supplementary material for: Deleterious variants in LTBP4 are associated with severe pediatric sepsis
Source: Pediatr Res. 2025 Oct 11;99(5):2007–18. doi: 10.1038/s41390-025-04420-3 (PMC13182162; doi:10.1038/s41390-025-04420-3)
Supplement: Supplementary file 9 — S. Table 5 [file 41390_2025_4420_MOESM9_ESM.docx]

**S. Table 5. Biomarkers measured at day 1 by phenotype PedSep-B (N = 319)**

| **Biomarker^a^** | **PedSep-B (N = 86)** | **Non-PedSep-B (N = 233)** | **p-value** |
| --- | --- | --- | --- |
| ADAMTS13, % | 70.5 (56.2, 89.2) | 72.0 (53.8, 88.2) | 0.710 |
| SFasLg, pg/ml | 38.4 (29.9, 67.9) | 49.0 (31.9, 79.5) | 0.160 |
| Ex vivo TNF-α, pg/ml | 336.3 (99.2, 806.8) | 564.7 (184.3, 1049.2) | 0.022 |
| TNF-α, pg/ml | 1049.2 (1049.2, 1500.0) | 957.3 (602.2, 1049.2) | 0.023 |
| sCD163, pg/ml | 309800 (179839, 508713) | 276625 (177921, 427838) | 0.363 |
| IFN-β, pg/ml | 6.4 (6.4, 10.5) | 6.4 (6.4, 6.4) | 0.014 |
| IL-22, pg/ml | 28.0 (21.3, 36.0) | 24.8 (20.1, 34.2) | 0.166 |
| IL-18, pg/ml | 444.0 (258.8, 820.7) | 400.3 (250.4, 665.8) | 0.386 |
| IL-18BP, pg/ml | 16142 (8938, 27901) | 16000 (8699, 27428) | 0.920 |
| MIG/CXCL9, pg/ml | 772.7 (403.0, 2033.0) | 810.5 (458.8, 1993.6) | 0.658 |
| IL-1β, pg/ml | 2.8 (2.1, 3.3) | 2.8 (2.4, 3.3) | 0.984 |
| IL-4, pg/ml | 4.7 (3.9, 6.8) | 4.7 (3.5, 6.5) | 0.293 |
| IL-6, pg/ml | 8.9 (6.5, 22.3) | 8.4 (6.2, 17.1) | 0.583 |
| IL-8, pg/ml | 54.7 (34.7, 113.7) | 49.4 (30.1, 88.7) | 0.242 |
| IL-10, pg/ml | 22.4 (18.0, 37.7) | 21.7 (16.3, 32.8) | 0.322 |
| IL-13, pg/ml | 3.1 (3.1, 3.5) | 3.1 (3.1, 4.1) | 0.787 |
| IL-17A, pg/ml | 19.1 (16.5, 25.1) | 18.3 (15.6, 23.4) | 0.060 |
| IFN-γ, pg/ml | 2.8 (2.8, 3.0) | 2.8 (2.8, 2.8) | 0.127 |
| IP-10/CXCL10, pg/ml | 705.8 (257.2, 1723.9) | 769.1 (354.8, 2284.0) | 0.409 |
| MCP-1/CCL2, pg/ml | 169.9 (83.5, 377.0) | 130.8 (57.4, 300.8) | 0.159 |
| MIP-1α, pg/ml | 0.6 (0.6, 10.2) | 0.6 (0.6, 5.8) | 0.210 |
| MIP-1β, pg/ml | 43.4 (32.3, 70.1) | 46.7 (31.5, 66.7) | 0.978 |
| MCP-3, pg/ml | 92.4 (92.4, 166.0) | 92.4 (92.4, 147.8) | 0.821 |
| IFN-α2, pg/ml | 125.7 (112.6, 148.3) | 125.7 (105.8, 140.2) | 0.181 |
| IL-1α, pg/ml | 9.4 (9.4, 16.4) | 9.4 (9.4, 13.2) | 0.211 |
| IL-2RA, pg/ml | 401.8 (199.2, 689.9) | 364.7 (246.1, 561.6) | 0.520 |
| IL-3, pg/ml | 624.4 (529.0, 724.4) | 612.2 (512.5, 724.4) | 0.412 |
| IL-16, pg/ml | 605.0 (444.1, 759.8) | 575.9 (418.2, 778.0) | 0.752 |
| M-CSF, pg/ml | 30.6 (19.8, 54.6) | 28.1 (15.6, 50.8) | 0.250 |
| SCF, pg/ml | 152.8 (109.2, 229.5) | 154.2 (116.1, 232.2) | 0.680 |
| TRAIL, pg/ml | 35.4 (25.4, 51.3) | 39.1 (30.3, 53.5) | 0.204 |
| CRPH, mg/dL | 10.2 (6.0, 19.6) | 9.4 (2.9, 16.2) | 0.069 |
| Ferritin, ng/mL | 199.5 (111.0, 545.0) | 182.3 (88.6, 481.8) | 0.354 |

^a^ All biomarkers are measured one time concomitantly in the first day. Values in table are summarized as median (IQR)
