# Supplementary material for: Deleterious variants in LTBP4 are associated with severe pediatric sepsis
Source: Pediatr Res. 2025 Oct 11;99(5):2007–18. doi: 10.1038/s41390-025-04420-3 (PMC13182162; doi:10.1038/s41390-025-04420-3)
Supplement: Supplementary file 10 — S. Table 6 [file 41390_2025_4420_MOESM10_ESM.docx]

**S. Table 6. Biomarkers measured at day 1 by phenotype PedSep-C (N = 319)**

| **Biomarker^a^** | **PedSep-C (N = 77)** | **Non-PedSep-C (N = 242)** | **p-value** |
| --- | --- | --- | --- |
| ADAMTS13, % | 69.0 (47.0, 84.0) | 73.0 (57.0, 93.0) | 0.061 |
| SFasLg, pg/ml | 42.7 (28.2, 66.9) | 49.3 (33.0, 79.5) | 0.077 |
| Ex vivo TNF-α, pg/ml | 384.8 (78.2, 485.4) | 526.7 (230.7, 1049.2) | 0.021 |
| TNF-α, pg/ml | 938.9 (430.0, 1049.2) | 1049.2 (728.0, 1049.2) | 0.268 |
| sCD163, pg/ml | 334582 (206378, 489112) | 262850 (174207, 422271) | 0.028 |
| IFN-β, pg/ml | 6.4 (6.4, 8.2) | 6.4 (6.4, 8.2) | 0.946 |
| IL-22, pg/ml | 26.0 (20.1, 33.0) | 24.8 (20.1, 34.2) | 0.440 |
| IL-18, pg/ml | 510.7 (269.2, 918.1) | 391.5 (240.6, 638.6) | 0.012 |
| IL-18BP, pg/ml | 22755 (14543, 32016) | 13654 (7423, 26090) | <0.001 |
| MIG/CXCL9, pg/ml | 1145.3 (535.7, 2394.1) | 753.6 (428.4, 1781.8) | 0.037 |
| IL-1β, pg/ml | 2.8 (2.4, 3.3) | 2.8 (2.2, 3.2) | 0.339 |
| IL-4, pg/ml | 4.7 (3.5, 6.5) | 4.7 (3.5, 6.5) | 0.506 |
| IL-6, pg/ml | 10.3 (6.9, 28.4) | 8.1 (6.0, 16.4) | 0.009 |
| IL-8, pg/ml | 49.4 (30.5, 92.2) | 51.0 (31.4, 91.1) | 0.919 |
| IL-10, pg/ml | 22.5 (17.5, 37.0) | 21.7 (16.3, 30.6) | 0.232 |
| IL-13, pg/ml | 3.1 (3.1, 4.3) | 3.1 (3.1, 3.9) | 0.795 |
| IL-17A, pg/ml | 20.9 (17.4, 26.8) | 18.3 (15.6, 22.6) | 0.014 |
| IFN-γ, pg/ml | 2.8 (2.8, 2.8) | 2.8 (2.8, 3.0) | 0.288 |
| IP-10/CXCL10, pg/ml | 985.1 (412.6, 2704.1) | 668.5 (303.5, 1939.3) | 0.025 |
| MCP-1/CCL2, pg/ml | 169.1 (83.9, 455.5) | 125.8 (57.2, 290.6) | 0.060 |
| MIP-1α, pg/ml | 0.6 (0.6, 9.0) | 0.6 (0.6, 6.1) | 0.164 |
| MIP-1β, pg/ml | 48.4 (33.0, 79.6) | 45.5 (31.4, 64.5) | 0.167 |
| MCP-3, pg/ml | 119.5 (92.4, 166.0) | 92.4 (92.4, 147.8) | 0.430 |
| IFN-α2, pg/ml | 125.7 (105.8, 140.2) | 125.7 (105.8, 144.4) | 0.838 |
| IL-1α, pg/ml | 9.4 (9.4, 16.4) | 9.4 (9.4, 16.4) | 0.678 |
| IL-2RA, pg/ml | 367.7 (239.4, 696.7) | 380.6 (243.0, 575.4) | 0.844 |
| IL-3, pg/ml | 612.2 (529.0, 724.4) | 612.2 (529.0, 724.4) | 0.840 |
| IL-16, pg/ml | 563.6 (413.5, 743.3) | 590.2 (432.5, 795.6) | 0.391 |
| M-CSF, pg/ml | 31.9 (17.3, 54.7) | 28.1 (15.6, 51.1) | 0.269 |
| SCF, pg/ml | 148.9 (112.1, 216.6) | 154.2 (115.4, 233.8) | 0.397 |
| TRAIL, pg/ml | 35.4 (30.4, 45.4) | 39.1 (27.9, 56.4) | 0.336 |
| CRPH, mg/dL | 14.4 (9.2, 21.2) | 8.1 (2.6, 15.5) | <0.001 |
| Ferritin, ng/mL | 260.7 (165.0, 664.6) | 172.5 (87.1, 447.0) | <0.001 |

^a^ All biomarkers are measured one time concomitantly in the first day. Values in table are summarized as median (IQR)
