# Supplementary material for: Deleterious variants in LTBP4 are associated with severe pediatric sepsis
Source: Pediatr Res. 2025 Oct 11;99(5):2007–18. doi: 10.1038/s41390-025-04420-3 (PMC13182162; doi:10.1038/s41390-025-04420-3)
Supplement: Supplementary file 11 — S. Table 7 [file 41390_2025_4420_MOESM11_ESM.docx]

**S. Table 7. Biomarkers measured at day 1 by phenotype PedSep-D (N = 319)**

| **Biomarker^a^** | **PedSep-D (N = 40)** | **Non-PedSep-D (N = 279)** | **p-value** |
| --- | --- | --- | --- |
| ADAMTS13, % | 55.0 (38.0, 66.2) | 74.0 (58.0, 93.0) | <0.001 |
| SFasLg, pg/ml | 42.4 (32.0, 62.3) | 48.1 (31.2, 80.2) | 0.175 |
| Ex vivo TNF-α, pg/ml | 623.3 (187.7, 1049.2) | 476.6 (139.4, 1049.2) | 0.355 |
| TNF-α, pg/ml | 728.7 (602.0, 1049.2) | 1049.2 (726.5, 1161.9) | 0.074 |
| sCD163, pg/ml | 464600 (270060, 741798) | 267704 (174016, 417048) | <0.001 |
| IFN-β, pg/ml | 6.4 (6.4, 6.4) | 6.4 (6.4, 8.2) | 0.356 |
| IL-22, pg/ml | 34.2 (25.4, 59.0) | 24.8 (20.1, 33.0) | <0.001 |
| IL-18, pg/ml | 518.4 (344.6, 744.9) | 398.0 (236.1, 694.2) | 0.074 |
| IL-18BP, pg/ml | 30713 (18613, 40878) | 14388 (8034, 24711) | <0.001 |
| MIG/CXCL9, pg/ml | 2462.4 (695.9, 4430.3) | 753.6 (412.8, 1528.2) | <0.001 |
| IL-1β, pg/ml | 3.0 (2.6, 3.3) | 2.8 (2.3, 3.3) | 0.155 |
| IL-4, pg/ml | 4.3 (3.5, 6.3) | 4.7 (3.5, 6.5) | 0.360 |
| IL-6, pg/ml | 17.1 (8.4, 43.8) | 8.4 (6.2, 15.0) | <0.001 |
| IL-8, pg/ml | 113.3 (60.7, 316.4) | 46.5 (30.3, 77.8) | <0.001 |
| IL-10, pg/ml | 29.9 (24.8, 71.0) | 21.1 (16.3, 31.0) | <0.001 |
| IL-13, pg/ml | 3.1 (3.1, 3.4) | 3.1 (3.1, 4.3) | 0.626 |
| IL-17A, pg/ml | 19.1 (16.5, 23.0) | 18.3 (16.5, 23.4) | 0.592 |
| IFN-γ, pg/ml | 2.8 (2.8, 2.8) | 2.8 (2.8, 3.0) | 0.089 |
| IP-10/CXCL10, pg/ml | 960.5 (526.4, 3160.0) | 692.5 (315.9, 2007.7) | 0.041 |
| MCP-1/CCL2, pg/ml | 190.9 (107.7, 488.8) | 129.7 (56.6, 288.6) | 0.016 |
| MIP-1α, pg/ml | 5.4 (1.7, 13.9) | 0.6 (0.6, 5.7) | <0.001 |
| MIP-1β, pg/ml | 56.8 (47.9, 89.3) | 43.8 (30.6, 64.2) | 0.002 |
| MCP-3, pg/ml | 92.4 (92.4, 147.8) | 92.4 (92.4, 166.0) | 0.780 |
| IFN-α2, pg/ml | 120.0 (105.8, 140.2) | 125.7 (105.8, 144.4) | 0.522 |
| IL-1α, pg/ml | 9.4 (9.4, 14.8) | 9.4 (9.4, 16.4) | 0.784 |
| IL-2RA, pg/ml | 456.0 (347.0, 660.9) | 357.6 (223.9, 579.7) | 0.014 |
| IL-3, pg/ml | 612.2 (496.1, 708.7) | 612.2 (529.0, 724.4) | 0.428 |
| IL-16, pg/ml | 1146.9 (756.5, 1346.6) | 556.5 (416.4, 702.2) | <0.001 |
| M-CSF, pg/ml | 79.9 (46.2, 117.2) | 26.8 (15.4, 43.3) | <0.001 |
| SCF, pg/ml | 344.6 (226.2, 560.4) | 145.5 (111.1, 203.7) | <0.001 |
| TRAIL, pg/ml | 30.4 (25.4, 42.9) | 38.5 (30.4, 55.4) | 0.009 |
| CRPH, mg/dL | 10.1 (2.6, 20.7) | 9.6 (3.7, 16.3) | 0.767 |
| Ferritin, ng/mL | 575.0 (195.6, 1628.8) | 180.0 (87.3, 403.0) | <0.001 |

^a^ All biomarkers are measured one time concomitantly in the first day. Values in the table are summarized as median (IQR)
