# Supplementary material for: Deleterious variants in LTBP4 are associated with severe pediatric sepsis
Source: Pediatr Res. 2025 Oct 11;99(5):2007–18. doi: 10.1038/s41390-025-04420-3 (PMC13182162; doi:10.1038/s41390-025-04420-3)
Supplement: Supplementary file 12 — S. Table 8 [file 41390_2025_4420_MOESM12_ESM.docx]

**S. Table 8. Outcome by phenotype PedSep-D (N = 319)**

| **Outcome** | **PedSep-D** | **Non-PedSep-D** | **p-value** |
| --- | --- | --- | --- |
| Length of stay, median (IQR), d | 13 (5, 31) | 8 (5, 15) | 0.018 |
| Mortality, N (%) | 11 (24.5) | 17 (6.1) | < 0.001 |
| PICU free day, median (IQR), d | 11 (0, 21) | 21 (13, 25) | < 0.001 |
