# Supplementary material for: Deleterious variants in LTBP4 are associated with severe pediatric sepsis
Source: Pediatr Res. 2025 Oct 11;99(5):2007–18. doi: 10.1038/s41390-025-04420-3 (PMC13182162; doi:10.1038/s41390-025-04420-3)
Supplement: Supplementary file 13 — S. Table 9 [file 41390_2025_4420_MOESM13_ESM.docx]

# **S. Table 9. Single variant association and allele frequency for variants contributing to the gene-level significance.**

| **Gene** | **Variant** | **SNP Information^a^** | **Allele Frequency^b^** | | |
| --- | --- | --- | --- | --- | --- |
|  |  |  | **Black** | **White** | **Asian** |
| LTBP4 | rs370696272 | 19:41105311:C:T | 0.01187 | 0.00005504 | 0.000 |
|  | rs573310430 | 19:41122842:C:T | 0.00004134 | 0.000007788 | 0.000 |
|  | - | 19:41132970:C:T | - | - | - |
|  | rs200607327 | 19:41133005:G:A | 0.00004143 | 0.0006489 | 0.000 |
| PLA2G4E | - | 15:42276733:T:G | - | - | - |
|  | rs764494895 | 15:42278161:G:A | 0.000 | 0.00006737 | 0.000 |
|  | rs143966595 | 15:42293394:C:T | 0.0001654 | 0.0008021 | 0.000 |
|  | rs776016335 | 15:42298270:T:C | 0.000 | 0.000 | 0.00005561 |
| CCDC157 | rs9606721 | 22:30762035:A:G | 0.003390 | 0.02384 | 0.000 |
|  | rs540507025 | 22:30762080:C:T | 0.000 | 0.00002669 | 0.0004897 |
|  | rs143249037 | 22:30766366:G:A | 0.00004008 | 0.0007048 | 0.001535 |
|  | - | 22:30766438:C:A | - | - | - |
|  | rs139609945 | 22:30766496:C:T | 0.000 | 0.0001055 | 0.000 |
|  | rs1235664314 | 22:30766672:G:T | 0.0001147 | 0.000 | 0.000 |
|  | rs148283823 | 22:30766868:G:A | 0.006199 | 0.00008130 | 0.000 |
|  | rs202178544 | 22:30772567:T:C | 0.0004134 | 0.000007778 | 0.0001507 |

^a^SNPs are listed as chromosome: position (hg19): reference allele: alternative allele.

^b^Allele frequency of three populations according to gnomAD database.
