# Supplementary material for: Deleterious variants in LTBP4 are associated with severe pediatric sepsis
Source: Pediatr Res. 2025 Oct 11;99(5):2007–18. doi: 10.1038/s41390-025-04420-3 (PMC13182162; doi:10.1038/s41390-025-04420-3)
Supplement: Supplementary file 14 — S. Table 10 [file 41390_2025_4420_MOESM14_ESM.docx]

**S. Table 10. Comparison of p-values of tests comparing cytokines between rare variant carriers and non-carriers**

| **Biomarker** | **LTBP4** | **PLA2G4E** | **CCDC157** |
| --- | --- | --- | --- |
| ADAMTS13, % | 0.015 | 0.95 | 0.06 |
| SFasLg, pg/ml | 0.624 | 0.284 | 0.574 |
| Ex vivo TNF-α, pg/ml | 0.664 | 0.869 | 0.738 |
| TNF-α, pg/ml | 0.119 | 0.937 | 0.343 |
| sCD163, pg/ml | 0.812 | 0.703 | 0.852 |
| IFN-β, pg/ml | 0.131 | 0.534 | 0.22 |
| IL-22, pg/ml | 0.46 | 0.63 | 0.86 |
| IL-18, pg/ml | 0.903 | 0.25 | 0.767 |
| IL-18BP, pg/ml | 0.671 | 0.493 | 0.983 |
| MIG/CXCL9, pg/ml | 0.413 | 0.687 | 0.088 |
| IL-1β, pg/ml | 0.667 | 0.86 | 0.476 |
| IL-4, pg/ml | 0.025 | 0.186 | 0.035 |
| IL-6, pg/ml | 0.032 | 0.172 | 0.043 |
| IL-8, pg/ml | 0.286 | 0.396 | 0.044 |
| IL-10, pg/ml | 0.486 | 0.085 | 0.567 |
| IL-13, pg/ml | 0.315 | 0.801 | 0.952 |
| IL-17A, pg/ml | 0.097 | 0.152 | 0.089 |
| IFN-γ, pg/ml | 0.142 | 0.309 | 0.415 |
| IP-10/CXCL10, pg/ml | 0.715 | 0.319 | 0.059 |
| MCP-1/CCL2, pg/ml | 0.193 | 0.267 | 0.312 |
| MIP-1α, pg/ml | 0.087 | 0.361 | 0.184 |
| MIP-1β, pg/ml | 0.059 | 0.088 | 0.513 |
| MCP-3, pg/ml | 0.709 | 0.695 | 0.163 |
| IFN-α2, pg/ml | 0.418 | 0.73 | 0.94 |
| IL-1α, pg/ml | 0.846 | 0.741 | 0.287 |
| IL-2RA, pg/ml | 0.677 | 0.065 | 0.839 |
| IL-3, pg/ml | 0.825 | 0.819 | 0.24 |
| IL-16, pg/ml | 0.163 | 0.034 | 0.694 |
| M-CSF, pg/ml | 0.136 | 0.013 | 0.011 |
| SCF, pg/ml | 0.516 | 0.005 | 0.162 |
| TRAIL, pg/ml | 0.501 | 0.437 | 0.796 |
| CRPH, mg/dL | 0.189 | 0.022 | 0.658 |
| Ferritin, ng/mL | 0.023 | 0.07 | 0.652 |
