# Supplementary material for: Deleterious variants in LTBP4 are associated with severe pediatric sepsis
Source: Pediatr Res. 2025 Oct 11;99(5):2007–18. doi: 10.1038/s41390-025-04420-3 (PMC13182162; doi:10.1038/s41390-025-04420-3)
Supplement: Supplementary file 15 — S. Table 11 [file 41390_2025_4420_MOESM15_ESM.docx]

**S. Table 11. Median cytokine levels of rare variant carriers and non-carriers**

| **Biomarker** | **LTBP4** | | **PLA2G4E** | | **CCDC157** | |
| --- | --- | --- | --- | --- | --- | --- |
|  | **carriers** | **Non-carriers** | **carriers** | **No-carriers** | **carriers** | **Non-carriers** |
| ADAMTS13, % | 42.50 | 71.50 | 64.50 | 71.00 | 61.00 | 72.00 |
| SFasLg, pg/ml | 44.21 | 47.92 | 58.03 | 47.30 | 42.47 | 48.12 |
| Ex vivo TNF-α, pg/ml | 658.97 | 484.30 | 420.97 | 484.30 | 312.50 | 490.30 |
| TNF-α, pg/ml | 101.20 | 74.90 | 97.75 | 74.90 | 93.55 | 74.90 |
| sCD163, pg/ml | 425278.00 | 280412.00 | 265204.00 | 283880.00 | 327821.00 | 280412.00 |
| IFN-β, pg/ml | 6.40 | 6.40 | 6.40 | 6.40 | 6.40 | 6.40 |
| IL-22, pg/ml | 31.85 | 24.80 | 24.20 | 25.40 | 23.60 | 25.40 |
| IL-18, pg/ml | 397.90 | 411.20 | 504.40 | 408.90 | 398.00 | 412.80 |
| IL-18BP, pg/ml | 29541.00 | 15477.00 | 33202.00 | 15751.00 | 17799.00 | 15751.00 |
| MIG/CXCL9, pg/ml | 1482.00 | 779.00 | 2035.80 | 791.70 | 501.60 | 807.60 |
| IL-1β, pg/ml | 2.95 | 2.80 | 2.90 | 2.80 | 2.95 | 2.80 |
| IL-4, pg/ml | 4.90 | 4.70 | 5.05 | 4.70 | 4.30 | 4.70 |
| IL-6, pg/ml | 16.60 | 8.40 | 6.65 | 8.60 | 17.10 | 8.40 |
| IL-8, pg/ml | 99.80 | 49.80 | 83.15 | 50.60 | 81.55 | 49.40 |
| IL-10, pg/ml | 27.40 | 21.70 | 18.25 | 21.70 | 33.10 | 21.70 |
| IL-13, pg/ml | 3.10 | 3.10 | 3.10 | 3.10 | 3.10 | 3.10 |
| IL-17A, pg/ml | 16.95 | 18.70 | 19.55 | 18.30 | 19.55 | 18.30 |
| IFN-γ, pg/ml | 2.80 | 2.80 | 2.80 | 2.80 | 2.80 | 2.80 |
| IP-10/CXCL10, pg/ml | 1042.50 | 716.70 | 492.60 | 753.30 | 819.20 | 726.60 |
| MCP-1/CCL2, pg/ml | 131.90 | 133.30 | 248.30 | 133.35 | 220.80 | 131.55 |
| MIP-1α, pg/ml | 5.05 | 0.60 | 5.30 | 0.60 | 2.85 | 0.60 |
| MIP-1β, pg/ml | 73.25 | 45.10 | 53.85 | 45.45 | 55.30 | 45.10 |
| MCP-3, pg/ml | 92.40 | 92.40 | 92.40 | 92.40 | 147.80 | 92.40 |
| IFN-α2, pg/ml | 115.80 | 125.70 | 135.60 | 125.70 | 128.30 | 125.70 |
| IL-1α, pg/ml | 9.65 | 9.40 | 11.30 | 9.40 | 9.90 | 9.40 |
| IL-2RA, pg/ml | 428.00 | 371.80 | 503.60 | 367.90 | 380.30 | 373.60 |
| IL-3, pg/ml | 624.40 | 612.20 | 636.00 | 612.20 | 572.60 | 612.20 |
| IL-16, pg/ml | 1015.20 | 575.90 | 1055.30 | 572.90 | 607.60 | 575.90 |
| M-CSF, pg/ml | 46.15 | 28.70 | 117.25 | 28.70 | 50.35 | 28.10 |
| SCF, pg/ml | 237.40 | 151.50 | 287.40 | 151.50 | 210.70 | 151.50 |
| TRAIL, pg/ml | 35.40 | 37.90 | 45.40 | 37.90 | 35.40 | 37.90 |
| CRPH, mg/dL | 16.58 | 9.73 | 1.34 | 10.11 | 9.24 | 10.02 |
| Ferritin, ng/mL | 463.90 | 187.00 | 463.50 | 187.70 | 321.60 | 187.00 |
