# Supplementary material for: Deleterious variants in LTBP4 are associated with severe pediatric sepsis
Source: Pediatr Res. 2025 Oct 11;99(5):2007–18. doi: 10.1038/s41390-025-04420-3 (PMC13182162; doi:10.1038/s41390-025-04420-3)
Supplement: Supplementary file 16 — S. Table 12 [file 41390_2025_4420_MOESM16_ESM.docx]

**S. Table 12. Significant Results from Mediation Analysis**

| **Gene** | **Biomarker** | **Indirect effect (p-value)** | **Direct effect**  **(p-value)** | **Total effect**  **(p-value)** | **Proportion mediated** |
| --- | --- | --- | --- | --- | --- |
| LTBP4 | ADAMTS13 | 0.10 (0.008) | 0.51 (0.004) | 0.61 (< 0.001) | 0.16 |
| PLA2G4E | IL-16 | 0.12 (0.028) | 0.41 (0.016) | 0.53 (0.002) | 0.22 |
| CCDC157 | IL-8 | 0.16 (< 0.001) | 0.23 (0.008) | 0.40 (0.002) | 0.41 |
