# Supplementary material for: Deleterious variants in LTBP4 are associated with severe pediatric sepsis
Source: Pediatr Res. 2025 Oct 11;99(5):2007–18. doi: 10.1038/s41390-025-04420-3 (PMC13182162; doi:10.1038/s41390-025-04420-3)
Supplement: Supplementary file 17 — S. Table 13 [file 41390_2025_4420_MOESM17_ESM.docx]

**S. Table 13. P-values comparing rare variant carriers with non-carriers**

|  | **LTBP4** | **PLA2G4E** | **CCDC157** |
| --- | --- | --- | --- |
| Age, median (IQR), y | 0.453 | 0.042 | 0.673 |
| Sex, N (%) | 0.936 | 0.512 | 0.931 |
| Race, N (%) | 0.177 | 0.491 | 0.809 |
| Ethnicity, N (%) | 0.664 | 0.471 | 0.245 |
| Previous healthy | 0.635 | 1.000 | 1.000 |
| Immunocompromised, N (%) | 1.000 | 1.000 | 1.000 |
| PRISM Score, median (IQR) | 0.380 | 0.021 | 0.960 |
| OFI, median (IQR) | 0.016 | 0.384 | 0.009 |
| Infection, N (%) | 0.083 | 0.321 | 0.400 |
| Mortality, N (%) | 0.150 | 0.426 | 1.000 |

The tests were performed using the Kruskal–Wallis test, the χ2 test, or the Fisher’s exact test
