# Supplementary material for: Deleterious variants in LTBP4 are associated with severe pediatric sepsis
Source: Pediatr Res. 2025 Oct 11;99(5):2007–18. doi: 10.1038/s41390-025-04420-3 (PMC13182162; doi:10.1038/s41390-025-04420-3)
Supplement: Supplementary file 18 — Supplementary [file 41390_2025_4420_MOESM18_ESM.docx]

**Pathway-based tests suggest a potential role of metabolic processes in pediatric sepsis**

To provide insights into the genetic architecture of sepsis phenotypes beyond those obtained from single-gene association analysis, we ran GAUSS analysis based on gene-based test results, given that GAUSS detects gene set-phenotype association by aggregating not only genes of exome-wide significance but also genes with weak to moderate association signals. Although the GAUSS pathway-based association test did not identify significantly associated pathways passing the stringent multiple testing burden of the genome-level analysis (Table 5), top-ranked pathways in the pathway-level significance implicated a phospholipid metabolic processes (specifically, the N-acylphosphatidylethanolamine metabolic process driven by *PLA2G4E*), growth hormone secretion driven by *LTBP4*, endocytic recycling driven by *PLA2G4E*, and regulation of cilium beat frequency driven by *MKKS*, *CATSPER1*, *CCDC39*, *BBS2*, *GAS2L2*, and *DNAH11*.
