# Supplementary material for: Deleterious variants in LTBP4 are associated with severe pediatric sepsis
Source: Pediatr Res. Author manuscript; Available in PMC 2026 May 19. (PMC13182162; doi:10.1038/s41390-025-04420-3)
Supplement: Supplementary file 1 [file NIHMS2167558-supplement-Supplemental_Files.pdf]

S. Figure 1. Manhattan plot for PedSep-A (No. of genes = 3,846)

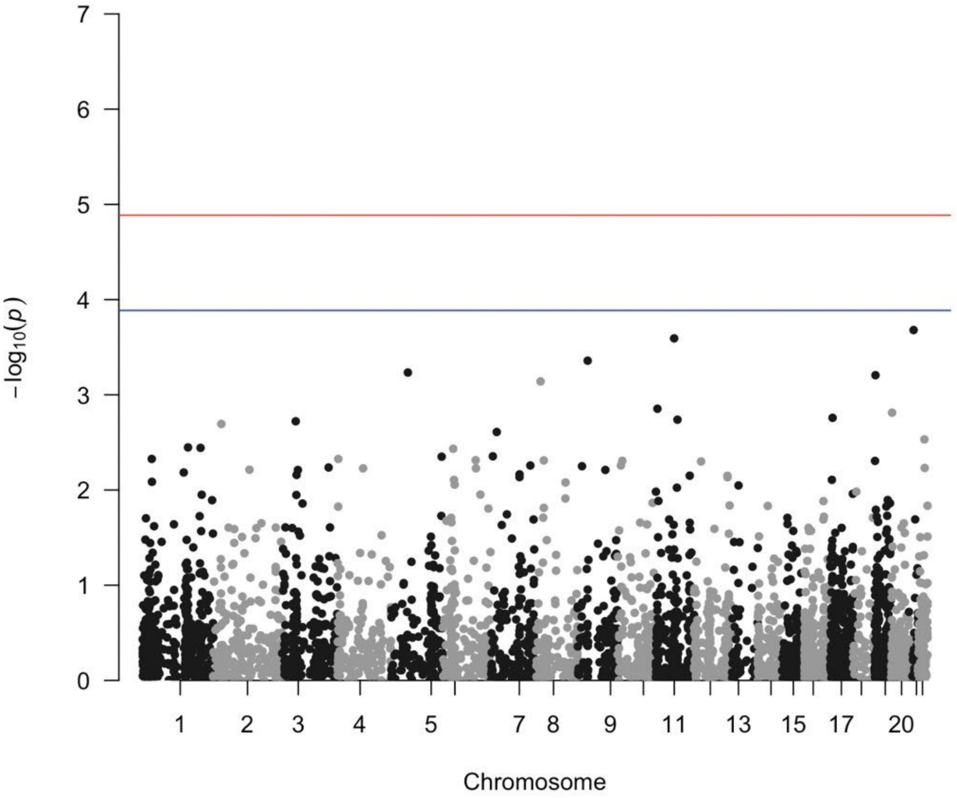

S. Figure 2. Manhattan plot for PedSep-B (No. of genes = 3,846)

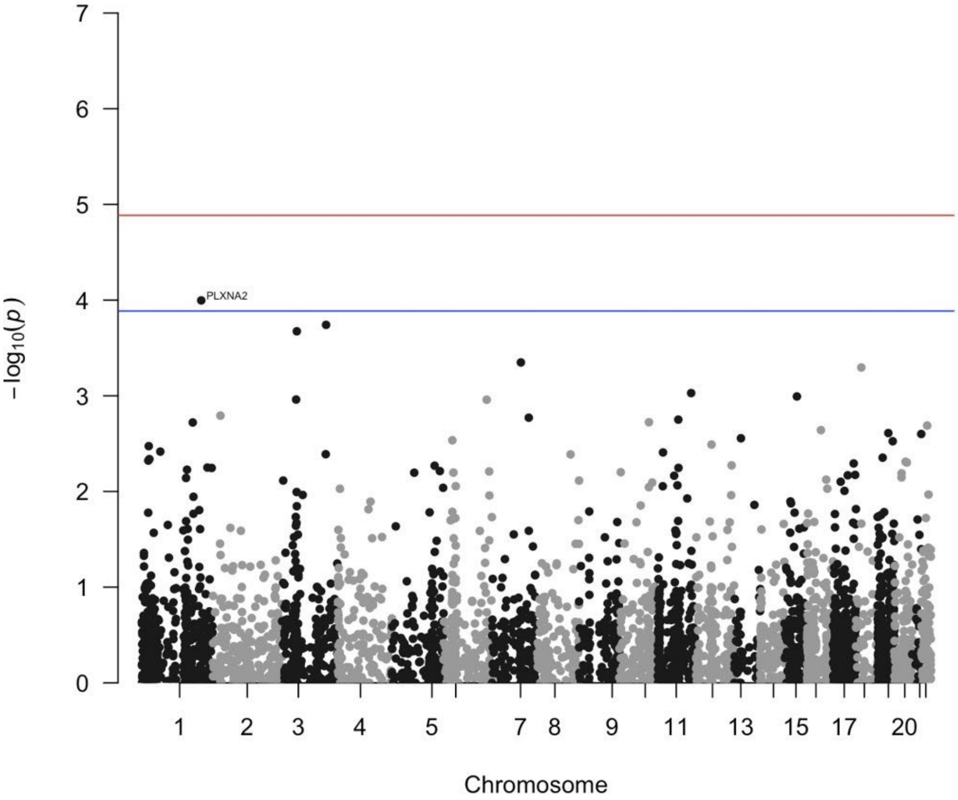

S. Figure 3. Manhattan plot for PedSep-C (No. of genes = 3,846)

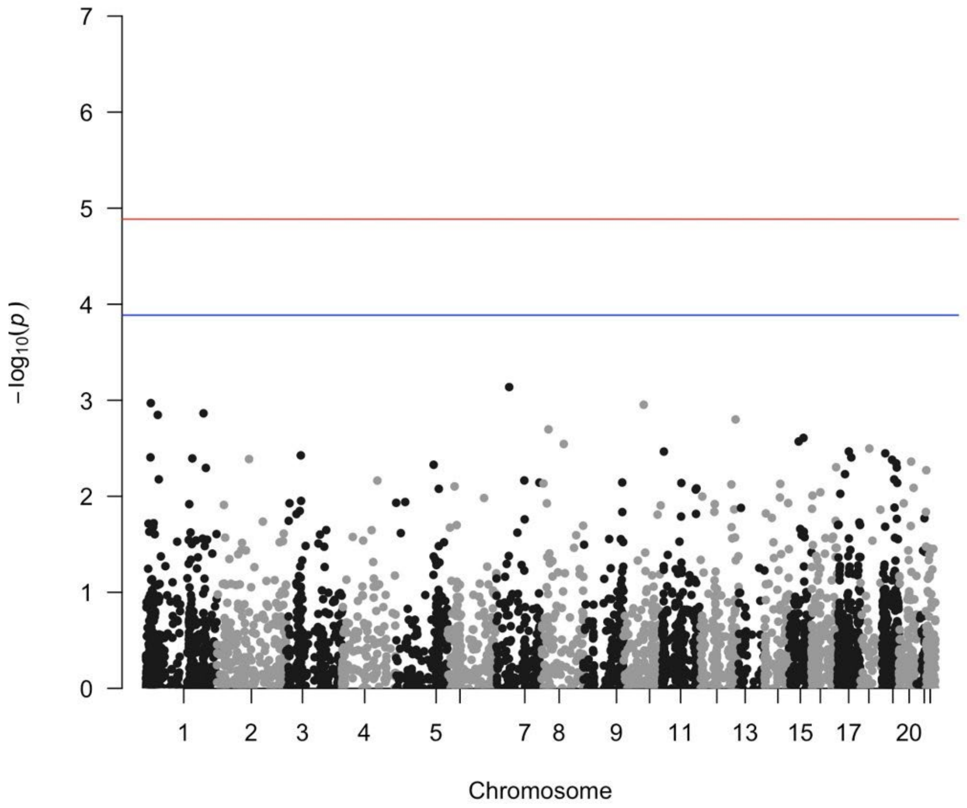

S. Figure 4. QQ plot of four phenotypes

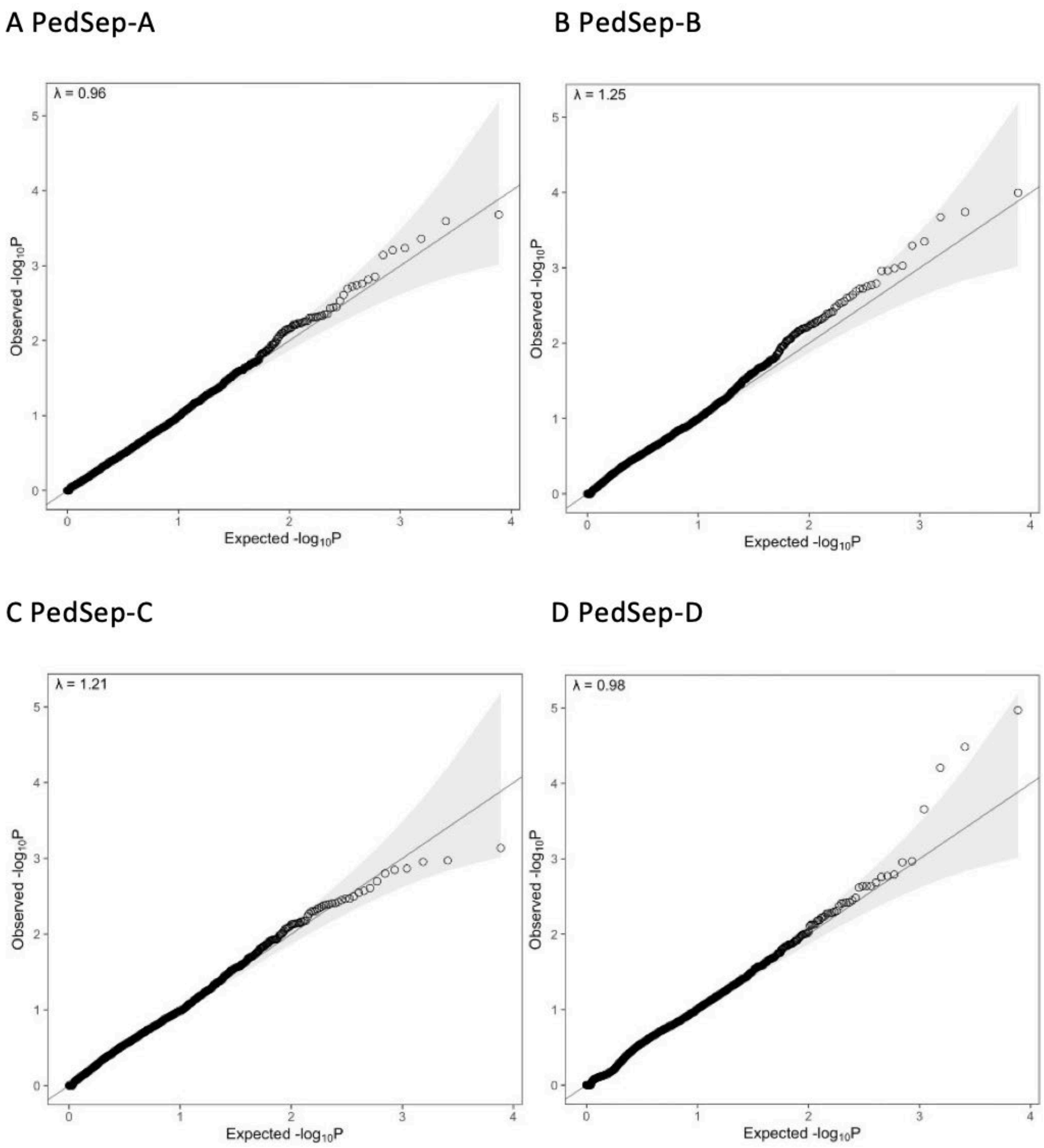

S. Table 1. Demographic and day 1 clinical characteristics of PedSep-A and Non-PedSep-A patients

Characteristics	PedSep-A	Non-PedSep-A	p-value[1]
**No. of Patients, *N* (%)**	116 (36.4)	203 (63.6)	
**Demographic**			
Age, years mean (SD)	3 (4)	9 (6)	<0.001
Male, N (%)	52 (44.8)	123 (60.6)	0.009
Hispanic, N (%)	24 (22)	26 (13.2)	0.056
Previous healthy, N (%)	79 (68.1)	74 (36.5)	<0.001
Surgery, N (%)	3 (2.6)	35 (17.2)	<0.001
**Organ Dysfunction**			
SIRS criteria[2], mean (SD)	2.9 (0.8)	2.9 (0.8)	0.900
OFI[3], mean (SD)	1.3 (0.5)	2.0 (0.9)	<0.001
**Inflammation**			
CRPH, mg/dL mean (SD)	7.4 (7.3)	14.1 (10.7)	<0.001
Low temperature, °C mean (SD)	36.7 (0.9)	36.5 (1.4)	0.639
High temperature, °C mean (SD)	37.8 (1.0)	37.8 (1.4)	0.912
ALC, /mm^3 median (IQR)	1.9 (1.3-3.4)	1.0 (0.5-1.7)	<0.001
Ferritin, ng/mL mean (IQR)	204.2 (71.5-210.5)	260.7 (130.7-681.6)	<0.001
**Pulmonary**			
Pulmonary OFI, N (%)	89 (76.7)	124 (61.1)	0.006
Intubation, N (%)	62 (53.4)	116 (57.1)	0.602
**Cardiovascular or Hemodynamic**			
Heart rate, bpm mean (SD)	168.6 (29.6)	147.1 (30.5)	<0.001
Systolic blood pressure, mmHg mean (SD)	85.1 (15.9)	79.2 (21.3)	0.007
CV OFI, N (%)	54 (46.6)	165 (81.3)	<0.001
**Renal**			
Creatinine, mg/dL median (IQR)	0.3 (0.2-0.4)	0.6 (0.4-1.2)	<0.001
Renal OFI, N (%)	0 (0.0)	26 (12.8)	<0.001
**Hepatic**			
Hepatic OFI, N (%)	3 (2.6)	28 (13.8)	<0.001
**Hematologic**			
Hemoglobin, g/dL mean (SD)	10.1 (1.8)	9.8 (2.0)	0.253
Platelets, K/mm^3 mean (SD)	257.2 (110.3)	134.7 (90.7)	<0.001
Hematologic OFI, N (%)	0 (0.0)	26 (12.8)	<0.001
**Other**			
Glasgow Coma Scale score[4,5], mean (SD)	8.4 (5.2)	8.3 (5.4)	0.840
CNS OFI, N (%)	10 (8.6)	32 (15.8)	0.100

IQR interquartile range, SIRS systemic inflammatory response syndrome, OFI organ failure index, ALC absolute lymphocyte count, CNS central nervous system

SI conversion factors: to convert alanine transaminase and aspartate aminotransferase to µkat/L, multiply by 0.0167; bilirubin to µmol/L, multiply by 17.104; C-reactive protein to nmol/L, multiply by 9.524; creatinine to µmol/L, multiply by 88.4

1 Comparisons across all 4 phenotypes were performed using the Kruskal–Wallis test, the $\chi 2$ test, or the Fisher's exact test

2 Indicates SIRS criteria ranging from 0 to 4 including abnormal heart rate, respiratory rate, temperature, and white blood cell count

3 OFI is an integer score reflecting the number of organ failures. Scores are either 0 or 1 for cardiovascular, hepatic, hematologic, respiratory, neurological, and renal, and summed for total range of 0 to 6. Cardiovascular, need for cardiovascular agent infusion support; Pulmonary, need for mechanical ventilation support with the ratio of the arterial partial pressure of oxygen and the fraction of inspired oxygen (PaO2/FiO2) < 300 without this support; Hepatic, total bilirubin > 1.0 mg/dL and alanine aminotransferase (ALT) > 100 units/L; Renal, serum creatinine > 1.0 mg/dL and oliguria (urine output < 0.5 mL/kg/h); Hematologic, thrombocytopenia < 100,000/mm3 and prothrombin time INR > 1.5 × normal; Central Nervous System, Glasgow Coma Scale (GCS) Score < 12 in the absence of sedatives

4 Corresponds to minimum or maximum value (as appropriate) within 6 h of hospital presentation

5 GCS ranges from 3 to 15

S. Table 2. Demographic and day 1 clinical characteristics of PedSep-B and Non-PedSep-B patients

Characteristics	PedSep-B	Non-PedSep-B	p-value[1]
**No. of Patients, *N* (%)**	86 (26.959)	233 (73.041)	
**Demographic**			
Age, years mean (SD)	8 (6)	6 (6)	0.064
Male, N (%)	56 (65.1)	119 (51.1)	0.035
Hispanic, N (%)	10 (12.2)	40 (17.9)	0.464
Previous healthy, N (%)	26 (30.2)	127 (54.5)	<0.001
Surgery, N (%)	18 (20.9)	20 (8.6)	0.005
**Organ Dysfunction**			
SIRS criteria[2], mean (SD)	3.0 (0.8)	2.9 (0.8)	0.401
OFI[3], mean (SD)	2.1 (0.6)	1.6 (0.9)	<0.001
**Inflammation**			
CRPH, mg/dL mean (SD)	13.5 (11.1)	11.0 (9.6)	0.064
Low temperature, °C mean (SD)	35.9 (1.7)	36.8 (0.9)	<0.001
High temperature, °C mean (SD)	37.3 (1.3)	38.0 (1.2)	<0.001
ALC, /mm^3 median (IQR)	1.1 (0.7-2.0)	1.3 (0.7-2.4)	0.395
Ferritin, ng/mL mean (IQR)	198.2 (111.6-535.2)	183.0 (89.0-481.7)	0.360
**Pulmonary**			
Pulmonary OFI, N (%)	73 (84.9)	140 (60.1)	<0.001
Intubation, N (%)	80 (93.0)	98 (42.1)	<0.001
**Cardiovascular or Hemodynamic**			
Heart rate, bpm mean (SD)	144.9 (29.4)	158.6 (32.0)	<0.001
Systolic blood pressure, mmHg mean (SD)	73.5 (22.0)	84.3 (17.9)	<0.001
CV OFI, N (%)	78 (90.7)	141 (60.5)	<0.001
**Renal**			
Creatinine, mg/dL median (IQR)	0.6 (0.3-0.8)	0.4 (0.3-0.8)	0.025
Renal OFI, N (%)	0 (0.0)	26 (11.2)	<0.001
**Hepatic**			
Hepatic OFI, N (%)	9 (10.5)	22 (9.4)	0.832
**Hematologic**			
Hemoglobin, g/dL mean (SD)	9.6 (2.1)	10.1 (1.9)	0.031
Platelets, K/mm^3 mean (SD)	155.7 (96.2)	187.9 (119.5)	0.033
Hematologic OFI, N (%)	0 (0.0)	26 (11.2)	<0.001
**Other**			
Glasgow Coma Scale score[4,5], mean (SD)	4.5 (3.3)	9.8 (5.2)	<0.001
CNS OFI, N (%)	19 (22.1)	23 (9.9)	0.007

IQR interquartile range, SIRS systemic inflammatory response syndrome, OFI organ failure index, ALC absolute lymphocyte count, CNS central nervous system

SI conversion factors: to convert alanine transaminase and aspartate aminotransferase to μkat/L, multiply by 0.0167; bilirubin to μmol/L, multiply by 17.104; C-reactive protein to nmol/L, multiply by 9.524; creatinine to μmol/L, multiply by 88.4

1 Comparisons across all 4 phenotypes were performed using the Kruskal–Wallis test, the χ^2 test, or the Fisher's exact test

2 Indicates SIRS criteria ranging from 0 to 4 including abnormal heart rate, respiratory rate, temperature, and white blood cell count

3 OFI is an integer score reflecting the number of organ failures. Scores are either 0 or 1 for cardiovascular, hepatic, hematologic, respiratory, neurological, and renal, and summed for total range of 0 to 6. Cardiovascular, need for cardiovascular agent infusion support; Pulmonary, need for mechanical ventilation support with the ratio of the arterial partial pressure of oxygen and the fraction of inspired oxygen (PaO_2/FiO_2) < 300 without this support; Hepatic, total bilirubin > 1.0 mg/dL and alanine aminotransferase (ALT) > 100 units/L; Renal, serum creatinine > 1.0 mg/dL and oliguria (urine output < 0.5 mL/kg/h); Hematologic, thrombocytopenia < 100,000/mm^3 and prothrombin time INR > 1.5 × normal; Central Nervous System, Glasgow Coma Scale (GCS) Score < 12 in the absence of sedatives

4 Corresponds to minimum or maximum value (as appropriate) within 6 h of hospital presentation

5 GCS ranges from 3 to 15

S. Table 3. Demographic and day 1 clinical characteristics of PedSep-C and Non-PedSep-C patients

Characteristics	PedSep-C	Non-PedSep-C	p-value[1]
**No. of Patients, *N* (%)**	77 (24.138)	242 (75.862)	
**Demographic**			
Age, years mean (SD)	10 (5)	6 (6)	<0.001
Male, N (%)	42 (54.5)	133 (55.0)	1.000
Hispanic, N (%)	13 (17.1)	37 (16.1)	0.409
Previous healthy, N (%)	31 (40.3)	122.0 (50.4)	0.155
Surgery, N (%)	9 (11.7)	29 (12.0)	1.000
**Organ Dysfunction**			
SIRS criteria[2], mean (SD)	2.8 (0.8)	2.9 (0.8)	0.200
OFI[3], mean (SD)	1.3 (0.5)	1.9 (0.9)	<0.001
**Inflammation**			
CRPH, mg/dL mean (SD)	15.4 (9.6)	10.5 (10.0)	<0.001
Low temperature, °C mean (SD)	37.2 (0.9)	36.4 (1.3)	<0.001
High temperature, °C mean (SD)	38.4 (1.3)	37.6 (1.2)	<0.001
ALC, /mm^3 median (IQR)	0.6 (0.3-1.1)	1.5 (1.0-2.7)	<0.001
Ferritin, ng/mL mean (IQR)	260.7 (165.0-3616.4)	173.5 (87.1-443.8)	<0.001
**Pulmonary**			
Pulmonary OFI, N (%)	26 (33.8)	187 (77.3)	<0.001
Intubation, N (%)	14 (18.2)	164.0 (67.8)	<0.001
**Cardiovascular or Hemodynamic**			
Heart rate, bpm mean (SD)	150.4 (26.7)	156.4 (33.3)	0.093
Systolic blood pressure, mmHg mean (SD)	85.7 (18.0)	80 (20.0)	0.080
CV OFI, N (%)	57 (74.0)	162 (66.9)	0.305
**Renal**			
Creatinine, mg/dL median (IQR)	0.5 (0.4-0.7)	0.4 (0.3-0.9)	0.090
Renal OFI, N (%)	0 (0.0)	26 (10.7)	0.001
**Hepatic**			
Hepatic OFI, N (%)	7 (9.1)	24 (9.9)	1.000
**Hematologic**			
Hemoglobin, g/dL mean (SD)	10.4 (1.9)	9.8 (1.9)	0.025
Platelets, K/mm^3 mean (SD)	137.6 (82.9)	192.5 (120.0)	<0.001
Hematologic OFI, N (%)	7 (9.1)	19 (7.9)	0.811
**Other**			
Glasgow Coma Scale score[4,5], mean (SD)	13.1 (3.2)	6.9 (5.0)	<0.001
CNS OFI, N (%)	4 (5.2)	38 (15.7)	0.029

IQR interquartile range, SIRS systemic inflammatory response syndrome, OFI organ failure index, ALC absolute lymphocyte count, CNS central nervous system

SI conversion factors: to convert alanine transaminase and aspartate aminotransferase to μkat/L, multiply by 0.0167; bilirubin to μmol/L, multiply by 17.104; C-reactive protein to nmol/L, multiply by 9.524; creatinine to μmol/L, multiply by 88.4

1 Comparisons across all 4 phenotypes were performed using the Kruskal–Wallis test, the χ^2 test, or the Fisher's exact test

2 Indicates SIRS criteria ranging from 0 to 4 including abnormal heart rate, respiratory rate, temperature, and white blood cell count

3 OFI is an integer score reflecting the number of organ failures. Scores are either 0 or 1 for cardiovascular, hepatic, hematologic, respiratory, neurological, and renal, and summed for total range of 0 to 6. Cardiovascular, need for cardiovascular agent infusion support; Pulmonary, need for mechanical ventilation support with the ratio of the arterial partial pressure of oxygen and the fraction of inspired oxygen (PaO_2/FiO_2) < 300 without this support; Hepatic, total bilirubin > 1.0 mg/dL and alanine aminotransferase (ALT) > 100 units/L; Renal, serum creatinine > 1.0 mg/dL and oliguria (urine output < 0.5 mL/kg/h); Hematologic, thrombocytopenia < 100,000/mm^3 and prothrombin time INR > 1.5 × normal; Central Nervous System, Glasgow Coma Scale (GCS) Score < 12 in the absence of sedatives

4 Corresponds to minimum or maximum value (as appropriate) within 6 h of hospital presentation

5 GCS ranges from 3 to 15

S. Table 4. Biomarkers measured at day 1 by phenotype PedSep-A (N = 319)

Biomarker[a]	PedSep-A (N = 116)	Non-PedSep-A (N = 203)	p-value
ADAMTS13, %	83.0 (65.0, 95.0)	66.0 (48.0, 83.5)	<0.001
SFasLg, pg/ml	59.8 (39.1, 87.0)	40.8 (29.4, 66.3)	<0.001
Ex vivo TNF-α, pg/ml	691.0 (334.0, 1049.2)	385.9 (97.1, 944.3)	<0.001
TNF-α, pg/ml	1049.2 (728.0, 1049.2)	1049.2 (604.1, 1049.2)	0.721
sCD163, pg/ml	212829 (155490, 300033)	340338 (215555, 580440)	<0.001
IFN-β, pg/ml	6.4 (6.4, 6.4)	6.4 (6.4, 8.2)	0.118
IL-22, pg/ml	22.4 (17.8, 29.5)	28.3 (22.1, 38.4)	<0.001
IL-18, pg/ml	329.6 (222.6, 533.6)	485.9 (294.6, 882.7)	<0.001
IL-18BP, pg/ml	9445 (6185, 16011)	20764 (12054, 33449)	<0.001
MIG/CXCL9, pg/ml	619.3 (406.0, 1061.2)	1026.0 (490.6, 2883.8)	<0.001
IL-1β, pg/ml	2.6 (2.1, 3.1)	2.8 (2.4, 3.3)	0.071
IL-4, pg/ml	4.7 (3.5, 6.3)	4.7 (3.5, 6.5)	0.799
IL-6, pg/ml	6.9 (5.8, 9.7)	10.9 (6.5, 30.2)	<0.001
IL-8, pg/ml	38.0 (26.4, 65.5)	59.5 (36.3, 130.5)	<0.001
IL-10, pg/ml	19.3 (15.4, 24.6)	25.0 (18.1, 40.3)	<0.001
IL-13, pg/ml	3.1 (3.1, 4.3)	3.1 (3.1, 3.5)	0.416
IL-17A, pg/ml	17.4 (15.1, 21.7)	20.0 (16.5, 25.1)	<0.001
IFN-γ, pg/ml	2.8 (2.8, 3.0)	2.8 (2.8, 2.8)	0.477
IP-10/CXCL10, pg/ml	492.2 (268.5, 1691.1)	850.5 (381.0, 2361.3)	0.008
MCP-1/CCL2, pg/ml	103.5 (48.3, 190.8)	178.6 (84.3, 400.5)	<0.001
MIP-1α, pg/ml	0.6 (0.6, 0.6)	2.0 (0.6, 10.7)	<0.001
MIP-1β, pg/ml	42.8 (28.1, 57.2)	50.7 (34.5, 81.1)	<0.001
MCP-3, pg/ml	92.4 (92.4, 147.8)	92.4 (92.4, 166.0)	0.269
IFN-α2, pg/ml	125.7 (105.8, 142.3)	125.7 (105.8, 142.8)	0.542
IL-1α, pg/ml	9.4 (9.4, 11.6)	9.4 (9.4, 16.4)	0.183
IL-2RA, pg/ml	343.5 (235.8, 504.6)	401.8 (243.4, 696.7)	0.014
IL-3, pg/ml	612.2 (496.1, 734.6)	612.2 (529.0, 724.4)	0.976
IL-16, pg/ml	544.1 (398.1, 660.5)	635.5 (453.6, 836.7)	<0.001
M-CSF, pg/ml	19.7 (13.8, 34.0)	36.2 (21.6, 78.6)	<0.001
SCF, pg/ml	138.2 (111.4, 192.7)	167.5 (115.4, 269.8)	<0.001
TRAIL, pg/ml	42.9 (32.9, 64.2)	35.4 (27.9, 48.5)	<0.001
CRPH, mg/dL	4.6 (1.3, 12.4)	11.4 (6.2, 20.8)	<0.001
Ferritin, ng/mL	121.0 (71.0, 204.5)	260.9 (130.6, 682.4)	<0.001

[a] All biomarkers are measured one time concomitantly in the first day. Values in table are summarized as median (IQR)

S. Table 5. Biomarkers measured at day 1 by phenotype PedSep-B (N = 319)

Biomarker[a]	PedSep-B (N = 86)	Non-PedSep-B (N = 233)	p-value
ADAMTS13, %	70.5 (56.2, 89.2)	72.0 (53.8, 88.2)	0.710
SFasLg, pg/ml	38.4 (29.9, 67.9)	49.0 (31.9, 79.5)	0.160
Ex vivo TNF-α, pg/ml	336.3 (99.2, 806.8)	564.7 (184.3, 1049.2)	0.022
TNF-α, pg/ml	1049.2 (1049.2, 1500.0)	957.3 (602.2, 1049.2)	0.023
sCD163, pg/ml	309800 (179839, 508713)	276625 (177921, 427838)	0.363
IFN-β, pg/ml	6.4 (6.4, 10.5)	6.4 (6.4, 6.4)	0.014
IL-22, pg/ml	28.0 (21.3, 36.0)	24.8 (20.1, 34.2)	0.166
IL-18, pg/ml	444.0 (258.8, 820.7)	400.3 (250.4, 665.8)	0.386
IL-18BP, pg/ml	16142 (8938, 27901)	16000 (8699, 27428)	0.920
MIG/CXCL9, pg/ml	772.7 (403.0, 2033.0)	810.5 (458.8, 1993.6)	0.658
IL-1β, pg/ml	2.8 (2.1, 3.3)	2.8 (2.4, 3.3)	0.984
IL-4, pg/ml	4.7 (3.9, 6.8)	4.7 (3.5, 6.5)	0.293
IL-6, pg/ml	8.9 (6.5, 22.3)	8.4 (6.2, 17.1)	0.583
IL-8, pg/ml	54.7 (34.7, 113.7)	49.4 (30.1, 88.7)	0.242
IL-10, pg/ml	22.4 (18.0, 37.7)	21.7 (16.3, 32.8)	0.322
IL-13, pg/ml	3.1 (3.1, 3.5)	3.1 (3.1, 4.1)	0.787
IL-17A, pg/ml	19.1 (16.5, 25.1)	18.3 (15.6, 23.4)	0.060
IFN-γ, pg/ml	2.8 (2.8, 3.0)	2.8 (2.8, 2.8)	0.127
IP-10/CXCL10, pg/ml	705.8 (257.2, 1723.9)	769.1 (354.8, 2284.0)	0.409
MCP-1/CCL2, pg/ml	169.9 (83.5, 377.0)	130.8 (57.4, 300.8)	0.159
MIP-1α, pg/ml	0.6 (0.6, 10.2)	0.6 (0.6, 5.8)	0.210
MIP-1β, pg/ml	43.4 (32.3, 70.1)	46.7 (31.5, 66.7)	0.978
MCP-3, pg/ml	92.4 (92.4, 166.0)	92.4 (92.4, 147.8)	0.821
IFN-α2, pg/ml	125.7 (112.6, 148.3)	125.7 (105.8, 140.2)	0.181
IL-1α, pg/ml	9.4 (9.4, 16.4)	9.4 (9.4, 13.2)	0.211
IL-2RA, pg/ml	401.8 (199.2, 689.9)	364.7 (246.1, 561.6)	0.520
IL-3, pg/ml	624.4 (529.0, 724.4)	612.2 (512.5, 724.4)	0.412
IL-16, pg/ml	605.0 (444.1, 759.8)	575.9 (418.2, 778.0)	0.752
M-CSF, pg/ml	30.6 (19.8, 54.6)	28.1 (15.6, 50.8)	0.250
SCF, pg/ml	152.8 (109.2, 229.5)	154.2 (116.1, 232.2)	0.680
TRAIL, pg/ml	35.4 (25.4, 51.3)	39.1 (30.3, 53.5)	0.204
CRPH, mg/dL	10.2 (6.0, 19.6)	9.4 (2.9, 16.2)	0.069
Ferritin, ng/mL	199.5 (111.0, 545.0)	182.3 (88.6, 481.8)	0.354

[a] All biomarkers are measured one time concomitantly in the first day. Values in table are summarized as median (IQR)

S. Table 6. Biomarkers measured at day 1 by phenotype PedSep-C (N = 319)

Biomarker[a]	PedSep-C (N = 77)	Non-PedSep-C (N = 242)	p-value
ADAMTS13, %	69.0 (47.0, 84.0)	73.0 (57.0, 93.0)	0.061
SFasLg, pg/ml	42.7 (28.2, 66.9)	49.3 (33.0, 79.5)	0.077
Ex vivo TNF-α, pg/ml	384.8 (78.2, 485.4)	526.7 (230.7, 1049.2)	0.021
TNF-α, pg/ml	938.9 (430.0, 1049.2)	1049.2 (728.0, 1049.2)	0.268
sCD163, pg/ml	334582 (206378, 489112)	262850 (174207, 422271)	0.028
IFN-β, pg/ml	6.4 (6.4, 8.2)	6.4 (6.4, 8.2)	0.946
IL-22, pg/ml	26.0 (20.1, 33.0)	24.8 (20.1, 34.2)	0.440
IL-18, pg/ml	510.7 (269.2, 918.1)	391.5 (240.6, 638.6)	0.012
IL-18BP, pg/ml	22755 (14543, 32016)	13654 (7423, 26090)	<0.001
MIG/CXCL9, pg/ml	1145.3 (535.7, 2394.1)	753.6 (428.4, 1781.8)	0.037
IL-1β, pg/ml	2.8 (2.4, 3.3)	2.8 (2.2, 3.2)	0.339
IL-4, pg/ml	4.7 (3.5, 6.5)	4.7 (3.5, 6.5)	0.506
IL-6, pg/ml	10.3 (6.9, 28.4)	8.1 (6.0, 16.4)	0.009
IL-8, pg/ml	49.4 (30.5, 92.2)	51.0 (31.4, 91.1)	0.919
IL-10, pg/ml	22.5 (17.5, 37.0)	21.7 (16.3, 30.6)	0.232
IL-13, pg/ml	3.1 (3.1, 4.3)	3.1 (3.1, 3.9)	0.795
IL-17A, pg/ml	20.9 (17.4, 26.8)	18.3 (15.6, 22.6)	0.014
IFN-γ, pg/ml	2.8 (2.8, 2.8)	2.8 (2.8, 3.0)	0.288
IP-10/CXCL10, pg/ml	985.1 (412.6, 2704.1)	668.5 (303.5, 1939.3)	0.025
MCP-1/CCL2, pg/ml	169.1 (83.9, 455.5)	125.8 (57.2, 290.6)	0.060
MIP-1α, pg/ml	0.6 (0.6, 9.0)	0.6 (0.6, 6.1)	0.164
MIP-1β, pg/ml	48.4 (33.0, 79.6)	45.5 (31.4, 64.5)	0.167
MCP-3, pg/ml	119.5 (92.4, 166.0)	92.4 (92.4, 147.8)	0.430
IFN-α2, pg/ml	125.7 (105.8, 140.2)	125.7 (105.8, 144.4)	0.838
IL-1α, pg/ml	9.4 (9.4, 16.4)	9.4 (9.4, 16.4)	0.678
IL-2RA, pg/ml	367.7 (239.4, 696.7)	380.6 (243.0, 575.4)	0.844
IL-3, pg/ml	612.2 (529.0, 724.4)	612.2 (529.0, 724.4)	0.840
IL-16, pg/ml	563.6 (413.5, 743.3)	590.2 (432.5, 795.6)	0.391
M-CSF, pg/ml	31.9 (17.3, 54.7)	28.1 (15.6, 51.1)	0.269
SCF, pg/ml	148.9 (112.1, 216.6)	154.2 (115.4, 233.8)	0.397
TRAIL, pg/ml	35.4 (30.4, 45.4)	39.1 (27.9, 56.4)	0.336
CRPH, mg/dL	14.4 (9.2, 21.2)	8.1 (2.6, 15.5)	<0.001
Ferritin, ng/mL	260.7 (165.0, 664.6)	172.5 (87.1, 447.0)	<0.001

[a] All biomarkers are measured one time concomitantly in the first day. Values in table are summarized as median (IQR)

S. Table 7. Biomarkers measured at day 1 by phenotype PedSep-D (N = 319)

Biomarker[a]	PedSep-D (N = 40)	Non-PedSep-D (N = 279)	p-value
ADAMTS13, %	55.0 (38.0, 66.2)	74.0 (58.0, 93.0)	<0.001
SFasLg, pg/ml	42.4 (32.0, 62.3)	48.1 (31.2, 80.2)	0.175
Ex vivo TNF-α, pg/ml	623.3 (187.7, 1049.2)	476.6 (139.4, 1049.2)	0.355
TNF-α, pg/ml	728.7 (602.0, 1049.2)	1049.2 (726.5, 1161.9)	0.074
sCD163, pg/ml	464600 (270060, 741798)	267704 (174016, 417048)	<0.001
IFN-β, pg/ml	6.4 (6.4, 6.4)	6.4 (6.4, 8.2)	0.356
IL-22, pg/ml	34.2 (25.4, 59.0)	24.8 (20.1, 33.0)	<0.001
IL-18, pg/ml	518.4 (344.6, 744.9)	398.0 (236.1, 694.2)	0.074
IL-18BP, pg/ml	30713 (18613, 40878)	14388 (8034, 24711)	<0.001
MIG/CXCL9, pg/ml	2462.4 (695.9, 4430.3)	753.6 (412.8, 1528.2)	<0.001
IL-1β, pg/ml	3.0 (2.6, 3.3)	2.8 (2.3, 3.3)	0.155
IL-4, pg/ml	4.3 (3.5, 6.3)	4.7 (3.5, 6.5)	0.360
IL-6, pg/ml	17.1 (8.4, 43.8)	8.4 (6.2, 15.0)	<0.001
IL-8, pg/ml	113.3 (60.7, 316.4)	46.5 (30.3, 77.8)	<0.001
IL-10, pg/ml	29.9 (24.8, 71.0)	21.1 (16.3, 31.0)	<0.001
IL-13, pg/ml	3.1 (3.1, 3.4)	3.1 (3.1, 4.3)	0.626
IL-17A, pg/ml	19.1 (16.5, 23.0)	18.3 (16.5, 23.4)	0.592
IFN-γ, pg/ml	2.8 (2.8, 2.8)	2.8 (2.8, 3.0)	0.089
IP-10/CXCL10, pg/ml	960.5 (526.4, 3160.0)	692.5 (315.9, 2007.7)	0.041
MCP-1/CCL2, pg/ml	190.9 (107.7, 488.8)	129.7 (56.6, 288.6)	0.016
MIP-1α, pg/ml	5.4 (1.7, 13.9)	0.6 (0.6, 5.7)	<0.001
MIP-1β, pg/ml	56.8 (47.9, 89.3)	43.8 (30.6, 64.2)	0.002
MCP-3, pg/ml	92.4 (92.4, 147.8)	92.4 (92.4, 166.0)	0.780
IFN-α2, pg/ml	120.0 (105.8, 140.2)	125.7 (105.8, 144.4)	0.522
IL-1α, pg/ml	9.4 (9.4, 14.8)	9.4 (9.4, 16.4)	0.784
IL-2RA, pg/ml	456.0 (347.0, 660.9)	357.6 (223.9, 579.7)	0.014
IL-3, pg/ml	612.2 (496.1, 708.7)	612.2 (529.0, 724.4)	0.428
IL-16, pg/ml	1146.9 (756.5, 1346.6)	556.5 (416.4, 702.2)	<0.001
M-CSF, pg/ml	79.9 (46.2, 117.2)	26.8 (15.4, 43.3)	<0.001
SCF, pg/ml	344.6 (226.2, 560.4)	145.5 (111.1, 203.7)	<0.001
TRAIL, pg/ml	30.4 (25.4, 42.9)	38.5 (30.4, 55.4)	0.009
CRPH, mg/dL	10.1 (2.6, 20.7)	9.6 (3.7, 16.3)	0.767
Ferritin, ng/mL	575.0 (195.6, 1628.8)	180.0 (87.3, 403.0)	<0.001

[a] All biomarkers are measured one time concomitantly in the first day. Values in the table are summarized as median (IQR)

S. Table 8. Outcome by phenotype PedSep-D (N = 319)

Outcome	PedSep-D	Non-PedSep-D	p-value
Length of stay, median (IQR), d	13 (5, 31)	8 (5, 15)	0.018
Mortality, N (%)	11 (24.5)	17 (6.1)	< 0.001
PICU free day, median (IQR), d	11 (0, 21)	21 (13, 25)	< 0.001

S. Table 9. Single variant association and allele frequency for variants contributing to the gene-level significance.

Gene	Variant	SNP Information[a]	Allele Frequency[b]		
			Black	White	Asian
LTBP4	rs370696272	19:41105311:C:T	0.01187	0.00005504	0.000
	rs573310430	19:41122842:C:T	0.00004134	0.000007788	0.000
	-	19:41132970:C:T	-	-	-
	rs200607327	19:41133005:G:A	0.00004143	0.0006489	0.000
PLA2G4E	-	15:42276733:T:G	-	-	-
	rs764494895	15:42278161:G:A	0.000	0.00006737	0.000
	rs143966595	15:42293394:C:T	0.0001654	0.0008021	0.000
	rs776016335	15:42298270:T:C	0.000	0.000	0.00005561
CCDC157	rs9606721	22:30762035:A:G	0.003390	0.02384	0.000
	rs540507025	22:30762080:C:T	0.000	0.00002669	0.0004897
	rs143249037	22:30766366:G:A	0.00004008	0.0007048	0.001535
	-	22:30766438:C:A	-	-	-
	rs139609945	22:30766496:C:T	0.000	0.0001055	0.000
	rs1235664314	22:30766672:G:T	0.0001147	0.000	0.000
	rs148283823	22:30766868:G:A	0.006199	0.00008130	0.000
	rs202178544	22:30772567:T:C	0.0004134	0.000007778	0.0001507

[a]SNPs are listed as chromosome: position (hg19): reference allele: alternative allele.
[b]Allele frequency of three populations according to gnomAD database.

S. Table 10. Comparison of p-values of tests comparing cytokines between rare variant carriers and non-carriers

Biomarker	LTBP4	PLA2G4E	CCDC157
ADAMTS13, %	0.015	0.95	0.06
SFasLg, pg/ml	0.624	0.284	0.574
Ex vivo TNF-α, pg/ml	0.664	0.869	0.738
TNF-α, pg/ml	0.119	0.937	0.343
sCD163, pg/ml	0.812	0.703	0.852
IFN-β, pg/ml	0.131	0.534	0.22
IL-22, pg/ml	0.46	0.63	0.86
IL-18, pg/ml	0.903	0.25	0.767
IL-18BP, pg/ml	0.671	0.493	0.983
MIG/CXCL9, pg/ml	0.413	0.687	0.088
IL-1β, pg/ml	0.667	0.86	0.476
IL-4, pg/ml	0.025	0.186	0.035
IL-6, pg/ml	0.032	0.172	0.043
IL-8, pg/ml	0.286	0.396	0.044
IL-10, pg/ml	0.486	0.085	0.567
IL-13, pg/ml	0.315	0.801	0.952
IL-17A, pg/ml	0.097	0.152	0.089
IFN-γ, pg/ml	0.142	0.309	0.415
IP-10/CXCL10, pg/ml	0.715	0.319	0.059
MCP-1/CCL2, pg/ml	0.193	0.267	0.312
MIP-1α, pg/ml	0.087	0.361	0.184
MIP-1β, pg/ml	0.059	0.088	0.513
MCP-3, pg/ml	0.709	0.695	0.163
IFN-α2, pg/ml	0.418	0.73	0.94
IL-1α, pg/ml	0.846	0.741	0.287
IL-2RA, pg/ml	0.677	0.065	0.839
IL-3, pg/ml	0.825	0.819	0.24
IL-16, pg/ml	0.163	0.034	0.694
M-CSF, pg/ml	0.136	0.013	0.011
SCF, pg/ml	0.516	0.005	0.162
TRAIL, pg/ml	0.501	0.437	0.796
CRPH, mg/dL	0.189	0.022	0.658
Ferritin, ng/mL	0.023	0.07	0.652

S. Table 11. Median cytokine levels of rare variant carriers and non-carriers

Biomarker	LTBP4		PLA2G4E		CCDC157	
	carriers	Non-carriers	carriers	No-carriers	carriers	Non-carriers
ADAMTS13, %	42.50	71.50	64.50	71.00	61.00	72.00
SFasLg, pg/ml	44.21	47.92	58.03	47.30	42.47	48.12
Ex vivo TNF-α, pg/ml	658.97	484.30	420.97	484.30	312.50	490.30
TNF-α, pg/ml	101.20	74.90	97.75	74.90	93.55	74.90
sCD163, pg/ml	425278.00	280412.00	265204.00	283880.00	327821.00	280412.00
IFN-β, pg/ml	6.40	6.40	6.40	6.40	6.40	6.40
IL-22, pg/ml	31.85	24.80	24.20	25.40	23.60	25.40
IL-18, pg/ml	397.90	411.20	504.40	408.90	398.00	412.80
IL-18BP, pg/ml	29541.00	15477.00	33202.00	15751.00	17799.00	15751.00
MIG/CXCL9, pg/ml	1482.00	779.00	2035.80	791.70	501.60	807.60
IL-1β, pg/ml	2.95	2.80	2.90	2.80	2.95	2.80
IL-4, pg/ml	4.90	4.70	5.05	4.70	4.30	4.70
IL-6, pg/ml	16.60	8.40	6.65	8.60	17.10	8.40
IL-8, pg/ml	99.80	49.80	83.15	50.60	81.55	49.40
IL-10, pg/ml	27.40	21.70	18.25	21.70	33.10	21.70
IL-13, pg/ml	3.10	3.10	3.10	3.10	3.10	3.10
IL-17A, pg/ml	16.95	18.70	19.55	18.30	19.55	18.30
IFN-γ, pg/ml	2.80	2.80	2.80	2.80	2.80	2.80
IP-10/CXCL10, pg/ml	1042.50	716.70	492.60	753.30	819.20	726.60
MCP-1/CCL2, pg/ml	131.90	133.30	248.30	133.35	220.80	131.55
MIP-1α, pg/ml	5.05	0.60	5.30	0.60	2.85	0.60
MIP-1β, pg/ml	73.25	45.10	53.85	45.45	55.30	45.10
MCP-3, pg/ml	92.40	92.40	92.40	92.40	147.80	92.40
IFN-α2, pg/ml	115.80	125.70	135.60	125.70	128.30	125.70
IL-1α, pg/ml	9.65	9.40	11.30	9.40	9.90	9.40
IL-2RA, pg/ml	428.00	371.80	503.60	367.90	380.30	373.60
IL-3, pg/ml	624.40	612.20	636.00	612.20	572.60	612.20
IL-16, pg/ml	1015.20	575.90	1055.30	572.90	607.60	575.90
M-CSF, pg/ml	46.15	28.70	117.25	28.70	50.35	28.10
SCF, pg/ml	237.40	151.50	287.40	151.50	210.70	151.50
TRAIL, pg/ml	35.40	37.90	45.40	37.90	35.40	37.90
CRPH, mg/dL	16.58	9.73	1.34	10.11	9.24	10.02
Ferritin, ng/mL	463.90	187.00	463.50	187.70	321.60	187.00

S. Table 12. Significant Results from Mediation Analysis

Gene	Biomarker	Indirect effect (p-value)	Direct effect (p-value)	Total effect (p-value)	Proportion mediated
LTBP4	ADAMTS13	0.10 (0.008)	0.51 (0.004)	0.61 (< 0.001)	0.16
PLA2G4E	IL-16	0.12 (0.028)	0.41 (0.016)	0.53 (0.002)	0.22
CCDC157	IL-8	0.16 (< 0.001)	0.23 (0.008)	0.40 (0.002)	0.41

S. Table 13. P-values comparing rare variant carriers with non-carriers

	LTBP4	PLA2G4E	CCDC157
Age, median (IQR), y	0.453	0.042	0.673
Sex, N (%)	0.936	0.512	0.931
Race, N (%)	0.177	0.491	0.809
Ethnicity, N (%)	0.664	0.471	0.245
Previous healthy	0.635	1.000	1.000
Immunocompromised, N (%)	1.000	1.000	1.000
PRISM Score, median (IQR)	0.380	0.021	0.960
OFI, median (IQR)	0.016	0.384	0.009
Infection, N (%)	0.083	0.321	0.400
Mortality, N (%)	0.150	0.426	1.000

The tests were performed using the Kruskal–Wallis test, the $\chi 2$ test, or the Fisher's exact test

Pathway-based tests suggest a potential role of metabolic processes in pediatric sepsis

To provide insights into the genetic architecture of sepsis phenotypes beyond those obtained from single-gene association analysis, we ran GAUSS analysis based on gene-based test results, given that GAUSS detects gene set-phenotype association by aggregating not only genes of exome-wide significance but also genes with weak to moderate association signals. Although the GAUSS pathway-based association test did not identify significantly associated pathways passing the stringent multiple testing burden of the genome-level analysis (Table 5), top-ranked pathways in the pathway-level significance implicated a phospholipid metabolic processes (specifically, the N-acylphosphatidylethanolamine metabolic process driven by *PLA2G4E*), growth hormone secretion driven by *LTBP4*, endocytic recycling driven by *PLA2G4E*, and regulation of cilium beat frequency driven by *MKKS*, *CATSPER1*, *CCDC39*, *BBS2*, *GAS2L2*, and *DNAH11*.
